# Long-Term Renal Transplant Outcome in Patients With Posterior Urethral Valves. Prognostic Factors Related to Bladder Dysfunction Management

**DOI:** 10.3389/fped.2021.646923

**Published:** 2021-05-11

**Authors:** María Virginia Amesty, Claudia García-Vaz, Laura Espinosa, María José Martínez-Urrutia, Pedro López-Pereira

**Affiliations:** ^1^Department of Pediatric Urology, Hospital Universitario La Paz, Madrid, Spain; ^2^Department of Preventive Medicine and Public Health, Hospital Universitario La Paz, Madrid, Spain; ^3^Department of Pediatric Nephrology, Hospital Universitario La Paz, Madrid, Spain

**Keywords:** posterior urethral valves, pediatric renal transplant, bladder dysfunction, long-term renal transplant outcome, lower urinary tract obstruction

## Abstract

**Introduction:** To obtain a successful renal transplant (RT) outcome in patients with posterior urethral valves (PUV), it is necessary to accomplish an adequate bladder dysfunction treatment. Our aim was to determine prognostic factors related to bladder dysfunction management in long-term RT outcome in patients with PUV.

**Methods:** A retrospective review of patients with PUV who received a first RT after 1985 in our institution with at least 5 years of follow-up was performed. Variables analyzed included prenatal diagnosis, age of diagnosis, initial presentation and management, bladder dysfunction treatment, other surgical treatments, pre-transplant dialysis, age of transplantation, type of donor, immunosuppression regimen, vascular and urological complications, rejections episodes, and graft survival.

**Results:** Fifty-one patients were included in the analysis. Prenatal diagnosis was done in 37.3%. Median age of diagnosis was 0.30 (0–88) months. Initial presentation was vesicoureteral reflux (VUR) in 78% and obstructive ureterohydronefrosis in 35.3%. Initial management was valve ablation (29.4%), pyelo-ureterostomy (64.7%), and vesicostomy (5.9%). In 33.3%, a type of bladder dysfunction treatment was performed: 21.6% bladder augmentation (BA), 15.7% Mitrofanoff procedure, 17.6% anticholinergic drugs, and 27.5% clean intermittent catheterization (CIC). Pre-transplant dialysis was received by 66.7%. Transplantation was performed at 6.28 ± 5.12 years, 62.7% were cadaveric and 37.3% living-donor grafts. Acute rejection episodes were found in 23.6%. Urological complications included recurrent urinary tract infections (UTIs) (31.4%); native kidneys VUR (31.4%); graft VUR (45.1%); and ureteral obstruction (2%). Vascular complications occurred in 3.9%. Mean graft survival was 11.1 ± 6.9 years. Analyzing the prognostic factor that influenced graft survival, patients with had CIC or a Mitrofanoff procedure had a significant better long-term graft survival after 10 years of follow-up (*p* < 0.05), despite of the existence of more recurrent UTIs in them. A better graft survival was also found in living-donor transplants (*p* < 0.05). No significant differences were observed in long-term graft survival regarding native kidneys or graft VUR, BA, immunosuppression regimen, or post-transplant UTIs.

**Conclusion:** Optimal bladder dysfunction treatment, including CIC with or without a Mitrofanoff procedure, might result in better long-term graft survival in patients with PUV. These procedures were not related to a worse RT outcome in spite of being associated with more frequent UTIs.

## Introduction

Posterior urethral valves (PUV) are a major cause of end stage renal disease (ESRD) in the pediatric population and constitute one of the most common causes of pediatric renal transplant (RT) (15.3%) (1). Patients with PUV are associated with some kind of bladder dysfunction, which has been called “valve bladder” (2). Manifestations of bladder dysfunction in PUV are variable, ranging from high-pressure low-compliant bladders and overactive bladders to myogenic-failure high-capacity bladders (3). In spite of bladder dysfunction, it has been demonstrated that RT outcome in patients with PUV is comparable to patients transplanted due to a non-urological anomaly in the mid and long-term (4–10). These favorable outcomes in graft survival are due to an adequate bladder dysfunction treatment before and after transplantation, but there is no consensus about what is considered optimal bladder management.

The aim of this study was to determine the prognostic factors related to bladder dysfunction management in long-term renal transplant outcome and to attempt to identify the best strategies to improve graft survival in these patients.

## Materials and Methods

This study was approved by the ethical committee of our center. A retrospective review of patients who received an RT between 1985 and 2020 with the diagnosis of PUV in our institution was performed. Patients who had a graft failure within the first 15 days post-transplantation and a post-transplant follow-up lower than 5 years were excluded from the analysis. In patients who received more than one renal transplant, only the first graft was considered for the analysis.

Variables analyzed included **data related to the PUV disease**: prenatal diagnosis and treatment, age at diagnosis, initial presentation (vesicoureteral reflux, obstructive ureterohydronefrosis), initial management (valve ablation, ureterostomy, vesicostomy), bladder dysfunction management (urodynamic findings, anticholinergics, clean intermittent catheterization, bladder augmentation, Mitrofanoff procedure), other surgical treatments (ureteral reimplantation, endoscopic reflux treatment, nephrectomy), and age of end stage renal disease settlement. The other variables analyzed included **data related to the renal transplant**: pre-transplant dialysis, type of donor, immunosuppression regimen applied, vascular and urological complications, rejections episodes, and graft and patient survival. We studied these variables to identify a prognostic factor that may have influence on long-term graft survival.

Bladder dysfunction diagnosis was achieved by performing an initial pre-transplant urodynamic study in all patients. After transplantation, all patients had a close follow-up that included a renal ultrasound and a voiding diary associated with urine culture if presenting urinary tract infection (UTI) symptoms. In cases with an increase of dilation of the urinary tract in the renal ultrasound, alteration of the voiding diary or symptomatic UTI, a urodynamic study was achieved to confirm bladder function status.

The criteria used for bladder dysfunction management was as follows: Anticholinergics were indicated when patients presented an overactive bladder in the urodynamic studies or patients with a low-compliant bladder. Clean intermittent catheterization (CIC) was indicated in patients with a myogenic failure in the urodynamic studies with a post-void residual volume of >10%. The Mitrofanoff procedure was indicated in the same cases of CIC but when urethral catheterization was painful or difficult to the patient. Bladder augmentation (BA) was indicated in patients with a urodynamic study with a low-compliant bladder that had not responded to anticholinergic drugs or a detrusor Botox injection.

Data were analyzed using SPSS version 25 (SPSS Inc., Chicago, IL, USA). Categorical data were compared using the Chi-squared test. Continuous data were presented as median and standard deviation and compared using Student's *t*-test. Graft and patient survival were analyzed by actuarial methods. Differences between the Kaplan-Meier survival curves were tested by log-rank tests. Differences were considered statistically significant at *p*-values of <0.05.

## Results

From a total of 501 RTs, 65 patients had a primary diagnosis of PUV, and 51 patients met the inclusion criteria for the analysis. Prenatal diagnosis was found in 19 patients (37.3%), while two patients (3.9%) received prenatal treatment (fetoscopic valve ablation). Median age of diagnosis was 0.30 (0–88) months (mean age 4.1 ± 13.8 months). Initial vesicoureteral reflux (VUR) was found in 39 boys (78%) (62% bilateral, 16% unilateral) and initial obstructive ureterohydronefrosis (UHN) in 18 (35.3%) (21.6% bilateral, 13.7% unilateral). Initial treatment consisted in valve ablation in 15 cases (29.4%), pyelo-ureterostomy in 33 (64.7%), and vesicostomy in 3 (5.9%). Seventeen patients (33.3%) had lower urinary tract dysfunction with an altered pattern in the urodynamic studies: 11 (21.6%) low-compliant bladders, 4 (7.8%) myogenic failures, and 2 (3.9%) overactive bladders. The rest of the patients did not present a significant bladder dysfunction in the pre-transplant assessment that required a specific pre-transplant treatment nor in the follow-up. Bladder dysfunction treatment consisted of 11 (21.6%) BAs (nine with ureter and two with intestine); 14 (27.5%) CICs; 8 (15.7%) Mitrofanoff procedures (six with ureter, two with Supplementary Material); and nine (17.6%) cases with anticholinergic drugs. Combination of treatments were achieved in most of the patients: BA+ CIC in 2 patients, BA+ CIC + anticholinergic drugs in 2; BA+ CIC + Mitrofanoff procedure in 4; BA+ CIC + Mitrofanoff + anticholinergic drugs in 2; CIC + Mitrofanoff procedure + anticholinergic drugs in 2; and CIC + anticholinergic drugs in 1. Two patients received only anticholinergic drugs, and one patient only CIC. In nine boys (17.6%) ureteral reimplantation was performed, five (9.8%) received reflux endoscopic treatment, and in 39 (76.5%) nephrectomy was carried out (35.3% unilateral, 41.2% bilateral).

From the total of 51 patients, 34 (66.7%) received pre-transplant dialysis (17.6% peritoneal, 49% hemodialysis), with a mean time in dialysis of 7.1 ± 8.6 months. Thirty-two patients (62.7%) received cadaveric transplants and 19 (37.3%) received living-related donor transplants. Mean age of transplantation was 6.28 ± 5.12 years. Episodes of acute rejection were found in 12 patients (23.6%). Post-transplant urological complications included recurrent urinary tract infections (UTIs) in 16 cases (31.4%); VUR to native kidneys in 16 (31.4%); VUR to the kidney graft in 23 (45.1%); and 1 (2%) ureteral obstruction after catheter extraction. Vascular complications occurred in 2 patients (3.9%): 1 had venous thrombosis with graft recovery after graft immediate re-transplantation, and 1 had an arterial hemorrhage with resolution after surgical exploration. Regarding the immunosuppression regimen, 26 patients (51%) received the initial immunosuppression protocol that consisted of induction with basiliximab or antithymocyte globulin, and triple therapy with mycophenolate mofetil, cyclosporine, and steroids; and 25 patients (49%) received the current protocol in which the cyclosporine was substituted by tacrolimus.

**Figure 1:**
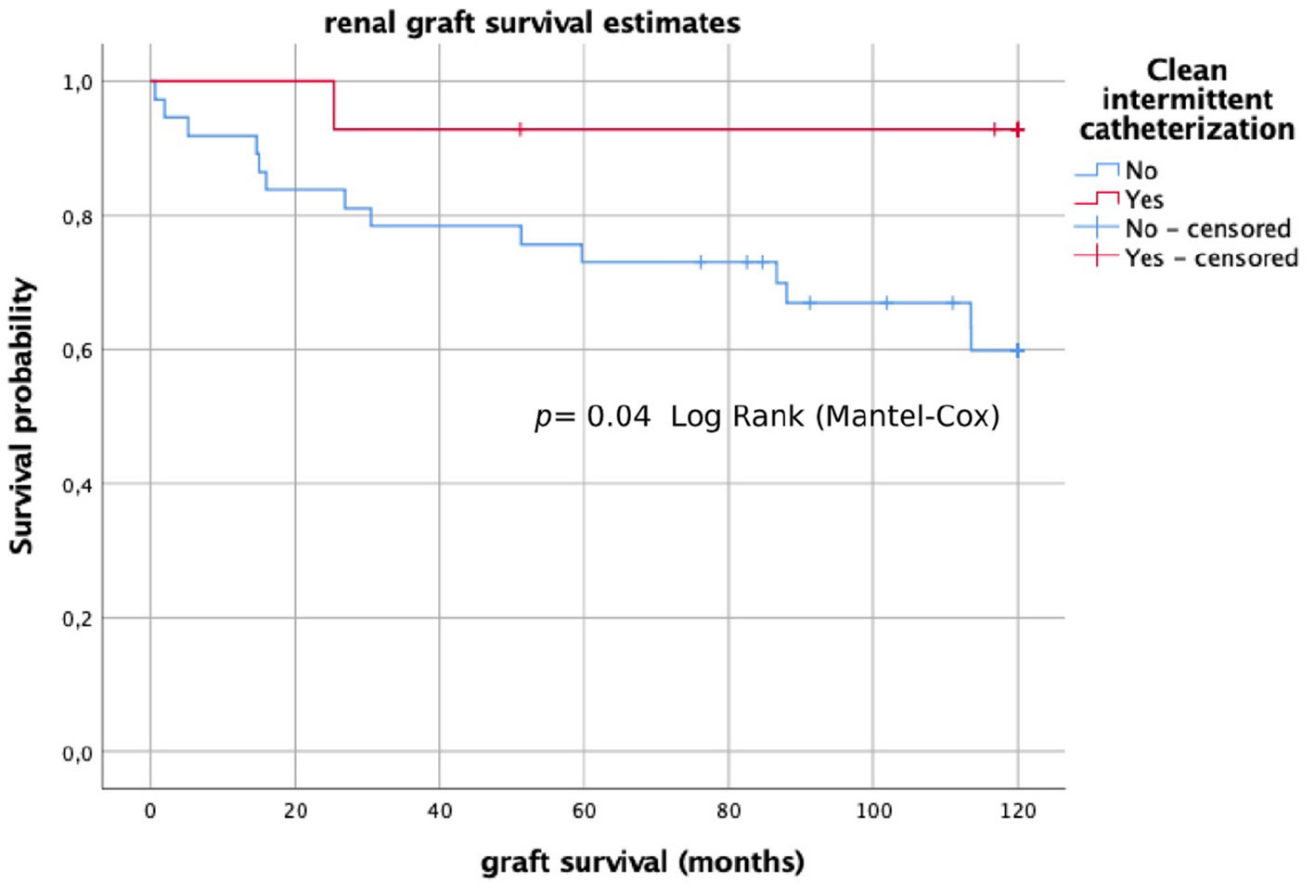
Long-term graft survival (Kaplan–Meier analysis) in patients with CIC and without CIC.

**Figure 2:**
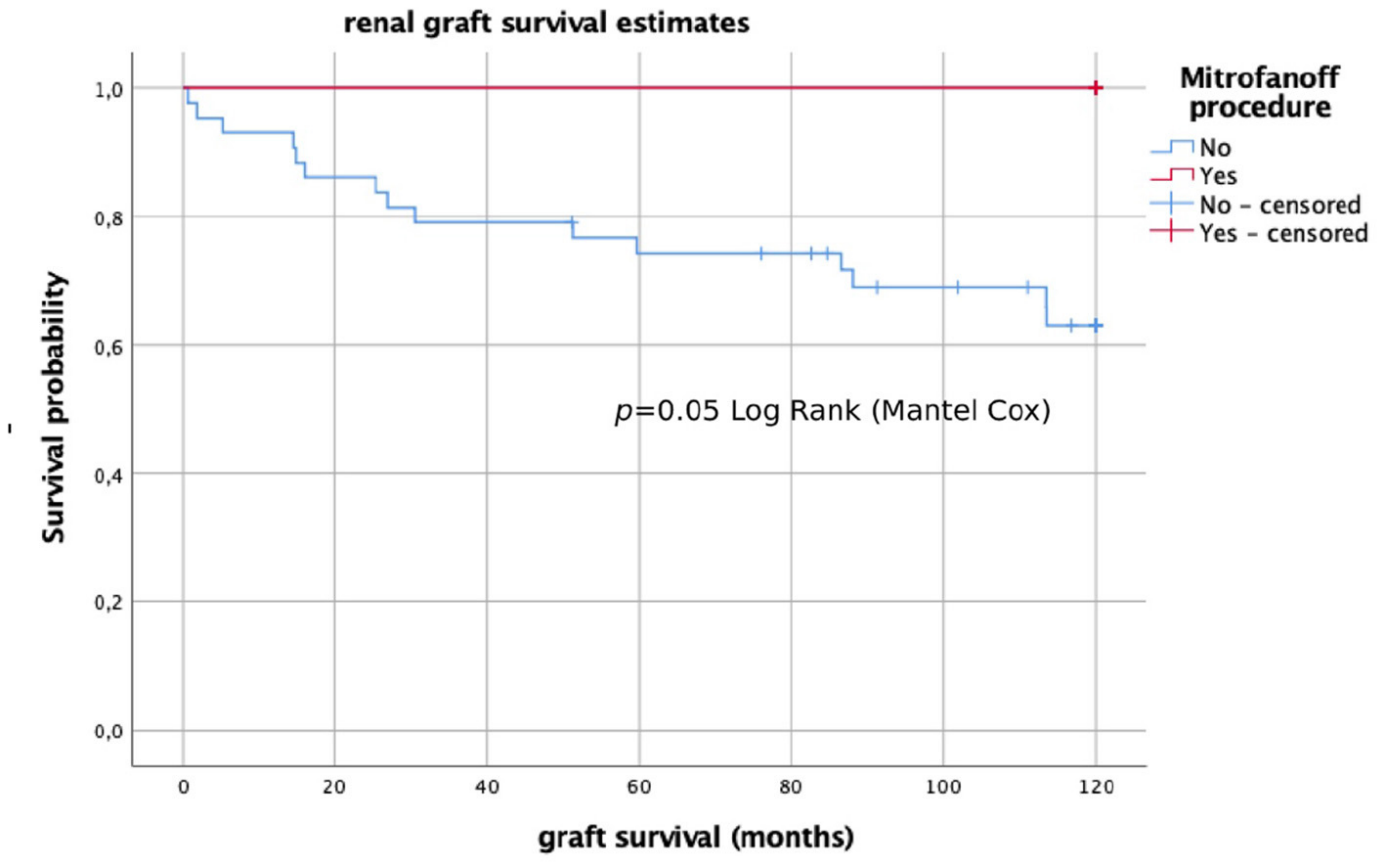
Long-term graft survival (Kaplan–Meier analysis) in patients who did and did not undergo the Mitrofanoff procedure.

During the follow-up 18 grafts were lost. Mean graft survival was 133.43 ± 83.35 months (11.1 ± 6.9 years). Causes of graft lost were chronic rejection in 14 (27.5%) cases, chronic glomerulopathy in 2 (3.9%), chronic toxicity to calcineurin inhibitors in 1 (2.0%), and death (with graft function) in 1 (2.0%). In analyzing the prognostic factors that may have an influence on long-term graft survival, we identified that patients who underwent CIC or had the Mitrofanoff procedure had a significantly better long-term graft survival after 10 years of follow-up (*p* = 0.05; *p* = 0.04) (Figures 1, 2). To analyze the possible confounders, we categorized patients into groups of CIC and no CIC patients, as well as Mitrofanoff and no Mitrofanoff patients, and we compared all the clinical variables collected in the study (Tables 1, 2). In these comparative analyses, we identified no significant differences in almost all the clinical variables, except for bladder dysfunction in urodynamics, bladder augmentation, anticholinergic drugs, and recurrent symptomatic UTIs. We found more cases of bladder dysfunction (100 vs. 8.1%), more cases with augmentation cystoplasty (71.4 vs. 2.7%; *p* = 0.00), more uses of anticholinergics (50 vs. 5.4%; *p* = 0.00), and more recurrent UTIs (85.7 vs. 10.8%; *p* = 0.00) in patients who underwent CIC compared to without CIC. We also found more cases of bladder dysfunction (100 vs. 20.9%), more cases of augmentation cystoplasty (75 vs. 11.6%; *p* = 0.00), more uses of anticholinergics (50 vs. 11.6%; *p* = 0.03), and more recurrent UTIs (87.5 vs. 20.9%; *p* = 0.00) in patients who had had the Mitrofanoff procedure compared to those who had not.

**Table 1:** Comparative analysis of clinical characteristics in patients with CIC and without CIC.

**Clinical variable**	**CIC (*n* = 14)**	**No CIC (*n* = 37)**	***P*-value**
Prenatal diagnosis	5 (35.7%)	14 (37.8%)	0.89
Prenatal treatment	1 (7.1%)	1 (2.7%)	0.48
Initial VUR	12 (85.7%)	27 (75%)	0.71
Initial obstructive UHN	4 (28.6%)	15 (42.9%)	0.35
Initial valve ablation (no urinary diversion)	3 (21.4%)	12 (32.4%)	0.51
Nephrectomy	13 (92.9%)	26 (72.3%)	0.22
Bladder dysfunction in the urodynamics	**14 (100%)**	**3 (8.1%)**	**0.00**
Bladder augmentation	**10 (71.4%)**	**1 (2.7%)**	**0.00**
Anticholinergic drugs	**7 (50%)**	**2 (5.4%)**	**0.00**
Mitrofanoff	**8 (57.1%)**	**0 (0%)**	**0.00**
Pre-transplant dialysis	8 (57.1%)	26 (70.3%)	0.51
Age of transplantation	7.24 ± 4.83	6.92 ± 4.90	0.84
Living-donor transplantation	5 (35.7%)	14 (37.8%)	1.00
Acute rejection episodes	2 (14.3%)	10 (27.1%)	0.55
Recurrent UTIs	**12 (85.7%)**	**4 (10.8%)**	**0.00**
VUR to native kidneys	5 (35.7%)	11 (32.4%)	0.89
VUR to the kidney graft	7 (53.8%)	16 (51.6%)	1.00
Other post-transplant urological complications	0 (0%)	1 (2.7%)	1.00
Post-transplant vascular complications	1 (7.1%)	1 (2.7%)	0.48
Initial immunosuppression protocol	8 (57.1%)	18 (48.6%)	0.59
Graft lost at the end of the study	**1 (7.1%)**	**17 (45.9%)**	**0.00**

*Bold values represent the clinical characteristics with a significant difference in the comparison*.

**Table 2:** Comparative analysis of clinical characteristics in patients who did and did not undergo the Mitrofanoff procedure.

**Clinical variable**	**Mitrofanoff (*n* = 8)**	**No Mitrofanoff (*n* = 43)**	***P*-value**
Prenatal diagnosis	3 (37.5%)	16 (37.2%)	1.00
Prenatal treatment	0 (0%)	2 (4.7%)	1.00
Initial VUR	8 (100%)	31 (73.8%)	0.17
Initial obstructive UHN	1 (12.5%)	18 (43.9%)	0.13
Initial valve ablation (no urinary diversion)	2 (25%)	13 (30.2%)	1.00
Nephrectomy	7 (87.5%)	32 (76.2%)	0.67
Bladder dysfunction in the urodynamics	**8 (100%)**	**9 (20.9%)**	**0.00**
Bladder augmentation	**6 (75%)**	**5 (11.6%)**	**0.00**
Anticholinergic drugs	**4 (50%)**	**5 (11.6%)**	**0.03**
Clean intermittent catheterization	**8 (100%)**	**6 (14%)**	**0.00**
Pre-transplant dialysis	4 (50%)	30 (69.8%)	0.42
Age of transplantation	6.90 ± 4.09	7.03 ± 5.00	0.94
Living-donor transplantation	4 (50%)	15 (34.9%)	0.45
Acute rejection episodes	2 (25%)	10 (23.6%)	0.92
Recurrent UTIs	**9 (87.5%)**	**7 (20.9%)**	**0.00**
VUR to native kidneys	5 (62.5%)	11 (27.5%)	0.10
VUR to the kidney graft	4 (57.1%)	19 (51.4%)	1.00
Other post-transplant urological complications	0 (0%)	1 (2.3%)	1.00
Post-transplant vascular complications	0 (0%)	2 (4.7%)	1.00
Initial immunosuppression protocol	4 (50%)	22 (51.2%)	1.00
Graft lost at the end of study	**1 (12.5%)**	**17 (39.53%)**	**0.01**

*Bold values represent the clinical characteristics with a significant difference in the comparison*.

**Figure 3:**
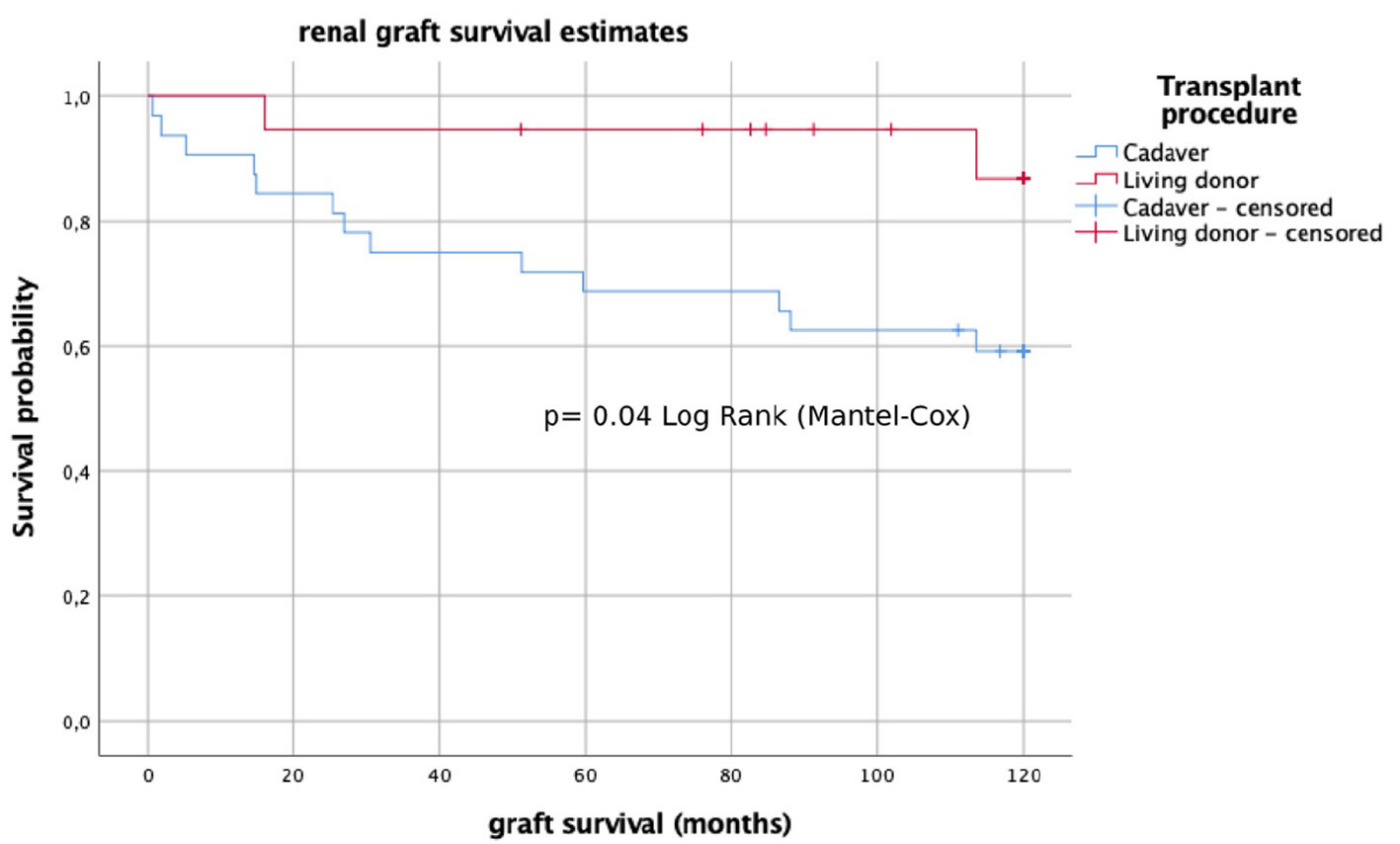
Long-term graft survival (Kaplan–Meier analysis) of living-donor and cadaveric transplants.

Another factor that influenced long-term graft survival was the type of transplant, with better graft survival in living-donors (*p* = 0.03) (Figure 3). We did not identify differences in long-term graft survival regarding other factors such as pre-transplant native kidneys VUR (*p* = 0.50), graft VUR (*p* = 0.86), BA (*p* = 0.47), pre-transplant dialysis (*p* = 0.51), immunosuppression regimen (*p* = 0.40), or post-transplant UTIs (*p* = 0.07).

Only one patient died due to septic shock secondary to a respiratory infection 60 months after transplantation.

## Discussion

RT is the therapy of choice in patients with ESRD (11). Most previous studies have demonstrated favorable RT outcomes in patients with PUV comparable to non-uropathic patients, with no major impact of bladder dysfunction associated with PUV in long-term graft survival (4–10). However, the surgical procedures that may be performed to optimize bladder function are still a controversial topic. Some authors have described better RT outcomes in patients with limited surgical procedures prior to transplantation (early vesicostomy and delay valve ablation, or primary valve ablation), and worse results in those patients with extensive surgical interventions (nephrostomy, pyelostomy, ureterostomy, urethral dilatation, ureteral reimplantation, or BA) (10, 12). On the other hand, Lopez Pereira et al. did not find any significant differences in RT outcome in patients with or without BA (13), and other studies found good RT outcomes in patients with augmented bladders (14–18). Also, Rickard et al. (19) identified similar RT outcomes in patients who did and did not undergo a Mitrofanoff procedure.

In the present study it has been found that patients with PUV that received a Mitrofanoff procedure or that accomplished CIC as treatments of their bladder dysfunction had a better long-term RT outcome. It is well-known that bladder dysfunction contributes to native renal function deterioration in patients with PUV (9, 20). Furthermore, bladder dysfunction characteristics tend to change over time as the patient grows, turning from overactive bladders in the first years of life to a myogenic failure pattern with an increase in post-voiding residuals (21, 22). It is probable that patients that accomplish CIC *via* urethra or *via* the Mitrofanoff procedure have fewer possibilities to develop these behavior changes and to have a better urodynamic pattern in the follow-up (9). Rickard et al. (19) found that implementing CIC *via* Mitrofanoff was associated with a delay in native renal function deterioration for a longer period, and that dialysis onset occurred at an older age compared to patients without Mitrofanoff. They postulated that this delay in renal failure was due to an improved compliance with CIC *via* Mitrofanoff and not *via* a sensate urethra. This argument may also be inferred to graft function, in which deterioration may be diminished with a better CIC compliance. However, in our study in most patients without Mitrofanoff and without CIC that had a graft failure, the cause of lost was mainly a chronic graft rejection. Despite the fact that bladder dysfunction was not identified as the main cause of the graft lost, it is probable that it may have contributed to renal function worsening in the very long-term. When analyzing the possible confounder factors, we did not identify any significant difference for most of the variables, except for BA, need of anticholinergic drugs, or recurrent UTIs, which were more frequent in the groups of CIC and Mitrofanoff procedure. These variables are considered unfavorable and we would have expected worse results in these group of patients. But we found the opposite, favorable results in these groups of patients with better long-term graft survival.

Regarding BA, in our study we did not find significant differences in long-term graft survival comparing patients with and without augmentation cystoplasty. This result is concordant with some previous studies about this topic (23). In the literature, there are not clear criteria to indicate BA in PUV patients (7, 14, 17, 24). Some authors recommend BA before RT to reduce the risk of graft function deterioration due to valve bladder syndrome, and also for technical reasons, to avoid the risk of graft pedicle lesions (14, 16). Other authors recommend performing BA after transplantation, because they argue that bladder dynamics may change after transplantation and BA may not be needed in some cases, also there is a potential increased risk of UTIs, and BA may preclude peritoneal dialysis (18, 25, 26). In our study, the number of patients that required BA was small. It may be due to the fact that early diagnosis and treatment of patients with posterior urethral valves in the recent years has diminished the need to performed a BA procedure, and that high-pressure low-compliant bladders treated early in life usually improve with an adequate CIC.

In respect of UTIs, no significant differences was found in 10-year graft survival in patients with and without post-transplant UTIs. But a tendency toward better results was found in patients with UTIs. This surprising finding could be explained by the fact that patients with UTIs had a closer follow-up with frequent reviews in the outpatient clinic, which may have optimized bladder dysfunction treatment and immunosuppression treatment. Several authors found increased prevalence of UTIs in transplanted patients with PUV, especially in patients with BA (13, 18, 27), but despite the increased risk of recurrent UTIs in these patients, most studies reported no direct contribution of UTIs in graft loss (10, 13, 23, 28, 29).

Another important finding of our study was that living-donor transplants achieved better long-term graft survival compared to cadaveric grafts. This is concordant with previous literature about this topic, in which it has been found that a living donation has a better RT outcome due to different reasons: this type of transplant involves better quality grafts, usually better cold ischemia times and better HLA matching, and allows for preemptive transplantation (30–34).

Limitations of the study include the retrospective nature of it and the small size of the patients analyzed. The groups of patients treated with CIC and the Mitrofanoff procedure were reduced and their favorable results could be obtained due to other confounding factors not identified in this study. Nevertheless, in spite of these limitations, some facts can be underlined: procedures to optimize bladder function in PUV, such as CIC (with or without the Mitrofanoff procedure) and BA, were not related to a worse long-term graft survival despite of being associated with more frequent UTIs. However, to confirm these findings, further multicenter studies with a higher number of patients must be undertaken.

## Conclusions

Optimal bladder dysfunction treatment, including CIC with or without a Mitrofanoff procedure, might result in a better long-term graft survival in patients with PUV. These procedures were not related to a worse RT outcome in spite of being associated with more frequent UTIs.

## Data Availability Statement

The raw data supporting the conclusions of this article will be made available by the authors, without undue reservation.

## Ethics Statement

The studies involving human participants were reviewed and approved by Comité de Ética Hospital Universitario La Paz. Written informed consent from the participants' legal guardian/next of kin was not required to participate in this study in accordance with the national legislation and the institutional requirements.

## Author Contributions

MA performed the study design, achieved the restrospective review of patients, and wrote the manuscript. CG-V performed the statistical analysis and help with the result interpretation and manuscript writing. LE helped to collect the data from the clinical charts of patients and helped in the manuscript writing. MM-U help to collect the data from the clinical charts and supervised the manuscript writing. PL-P is the senior and last author and he helped with the study design and supervised the manuscript writing. All authors discussed the results and commented on the manuscript.

## Conflict of Interest

The authors declare that the research was conducted in the absence of any commercial or financial relationships that could be construed as a potential conflict of interest.
