# Supplementary material for: Long-Term Renal Transplant Outcome in Patients With Posterior Urethral Valves. Prognostic Factors Related to Bladder Dysfunction Management
Source: Front Pediatr. 2021 May 11;9:646923. doi: 10.3389/fped.2021.646923 (PMC8144517; doi:10.3389/fped.2021.646923)
Supplement: Supplementary file 1 [file Data_Sheet_1.PDF]

ANÁLISIS DESCRIPTIVO: tablas de frecuencia de las distintas variables.

Diagnóstico prenatal

		Frecuencia	Porcentaje	Porcentaje válido	Porcentaje acumulado
Válido	No	32	62,7	62,7	62,7
	Sí	19	37,3	37,3	100,0
	Total	51	100,0	100,0	

Tratamiento intraútero

		Frecuencia	Porcentaje	Porcentaje válido	Porcentaje acumulado
Válido	No	49	96,1	96,1	96,1
	Ablación	2	3,9	3,9	100,0
	Total	51	100,0	100,0	

RVU inicial

		Frecuencia	Porcentaje	Porcentaje válido	Porcentaje acumulado
Válido	No	11	21,6	22,0	22,0
	Unilateral	8	15,7	16,0	38,0
	Bilateral	31	60,8	62,0	100,0
	Total	50	98,0	100,0	
Perdidos	Sistema	1	2,0		
Total		51	100,0		

UHN obstructiva inicial

		Frecuencia	Porcentaje	Porcentaje válido	Porcentaje acumulado
Válido	No	30	58,8	61,2	61,2
	Unilateral	7	13,7	14,3	75,5
	Bilateral	12	23,5	24,5	100,0
	Total	49	96,1	100,0	
Perdidos	Sistema	2	3,9		
Total		51	100,0		

Tratamiento inicial

		Frecuencia	Porcentaje	Porcentaje válido	Porcentaje acumulado
Válido	Resección	15	29,4	29,4	29,4
	Ureterostomía	33	64,7	64,7	94,1
	Vesicostomía	3	5,9	5,9	100,0
	Total	51	100,0	100,0	

Nefrectomía

		Frecuencia	Porcentaje	Porcentaje válido	Porcentaje acumulado
Válido	No	11	21,6	22,0	22,0
	Unilateral	18	35,3	36,0	58,0
	Bilateral	21	41,2	42,0	100,0
	Total	50	98,0	100,0	
Perdidos	Sistema	1	2,0		
Total		51	100,0		

Ampliación vesical

		Frecuencia	Porcentaje	Porcentaje válido	Porcentaje acumulado
Válido	No	40	78,4	78,4	78,4
	Sí	11	21,6	21,6	100,0
	Total	51	100,0	100,0	

Tipo ampliación

		Frecuencia	Porcentaje	Porcentaje válido	Porcentaje acumulado
Válido	No	39	76,5	78,0	78,0
	Uréter	9	17,6	18,0	96,0
	Íleon	2	3,9	4,0	100,0
	Total	50	98,0	100,0	
Perdidos	Sistema	1	2,0		
Total		51	100,0		

Anticolinérgicos

		Frecuencia	Porcentaje	Porcentaje válido	Porcentaje acumulado
Válido	No	42	82,4	82,4	82,4
	Sí	9	17,6	17,6	100,0
	Total	51	100,0	100,0	

CI

		Frecuencia	Porcentaje	Porcentaje válido	Porcentaje acumulado
Válido	No	37	72,5	72,5	72,5
	Sí	14	27,5	27,5	100,0
	Total	51	100,0	100,0	

Mitrofanoff

		Frecuencia	Porcentaje	Porcentaje válido	Porcentaje acumulado
Válido	No	43	84,3	84,3	84,3
	Sí	8	15,7	15,7	100,0
	Total	51	100,0	100,0	

Tipo Mitro

		Frecuencia	Porcentaje	Porcentaje válido	Porcentaje acumulado
Válido	No	43	84,3	84,3	84,3
	Uréter	6	11,8	11,8	96,1
	Apéndice	2	3,9	3,9	100,0
	Total	51	100,0	100,0	

RVU evol

		Frecuencia	Porcentaje	Porcentaje válido	Porcentaje acumulado
Válido	No	32	62,7	66,7	66,7
	Sí	16	31,4	33,3	100,0
	Total	48	94,1	100,0	
Perdidos	Sistema	3	5,9		
Total		51	100,0		

Grado RVU

		Frecuencia	Porcentaje	Porcentaje válido	Porcentaje acumulado
Válido	No	31	60,8	64,6	64,6
	Bajo	10	19,6	20,8	85,4
	Alto	7	13,7	14,6	100,0
	Total	48	94,1	100,0	
Perdidos	Sistema	3	5,9		
Total		51	100,0		

Tratamiento RVU

		Frecuencia	Porcentaje	Porcentaje válido	Porcentaje acumulado
Válido	No	36	70,6	72,0	72,0
	Reimplante	9	17,6	18,0	90,0
	Endoscópico	5	9,8	10,0	100,0
	Total	50	98,0	100,0	
Perdidos	Sistema	1	2,0		
Total		51	100,0		

RVU al injerto

		Frecuencia	Porcentaje	Porcentaje válido	Porcentaje acumulado
Válido	No	21	41,2	47,7	47,7
	Sí	23	45,1	52,3	100,0
	Total	44	86,3	100,0	
Perdidos	Sistema	7	13,7		
Total		51	100,0		

Grado RVU injerto

		Frecuencia	Porcentaje	Porcentaje válido	Porcentaje acumulado
Válido	No	20	39,2	46,5	46,5
	Bajo	17	33,3	39,5	86,0
	Alto	6	11,8	14,0	100,0
	Total	43	84,3	100,0	
Perdidos	Sistema	8	15,7		
Total		51	100,0		

Diálisis previa

		Frecuencia	Porcentaje	Porcentaje válido	Porcentaje acumulado
Válido	No	17	33,3	33,3	33,3
	Sí	34	66,7	66,7	100,0
	Total	51	100,0	100,0	

Tipo diálisis

		Frecuencia	Porcentaje	Porcentaje válido	Porcentaje acumulado
Válido	No	17	33,3	33,3	33,3
	Peritoneal	9	17,6	17,6	51,0
	Hemodiálisis	25	49,0	49,0	100,0
	Total	51	100,0	100,0	

Tipo trasplante

		Frecuencia	Porcentaje	Porcentaje válido	Porcentaje acumulado
Válido	Cadáver	32	62,7	62,7	62,7
	Vivo	19	37,3	37,3	100,0
	Total	51	100,0	100,0	

ITUs post-trasplante

		Frecuencia	Porcentaje	Porcentaje válido	Porcentaje acumulado
Válido	No	31	60,8	60,8	60,8
	1	3	5,9	5,9	66,7
	2	1	2,0	2,0	68,6
	Múltiples	16	31,4	31,4	100,0
	Total	51	100,0	100,0	

Número rechazos agudos

		Frecuencia	Porcentaje	Porcentaje válido	Porcentaje acumulado
Válido	0	39	76,5	76,5	76,5
	1	10	19,6	19,6	96,1
	3	1	2,0	2,0	98,0
	4	1	2,0	2,0	100,0
	Total	51	100,0	100,0	

Complicaciones urológicas

		Frecuencia	Porcentaje	Porcentaje válido	Porcentaje acumulado
Válido	No	50	98,0	98,0	98,0
	Sí	1	2,0	2,0	100,0
	Total	51	100,0	100,0	

Complicaciones vasculares

		Frecuencia	Porcentaje	Porcentaje válido	Porcentaje acumulado
Válido	No	49	96,1	96,1	96,1
	Sí	2	3,9	3,9	100,0
	Total	51	100,0	100,0	

Tipo inmunosupresión

		Frecuencia	Porcentaje	Porcentaje válido	Porcentaje acumulado
Válido	Cyc	26	51,0	51,0	51,0
	Azt	25	49,0	49,0	100,0
	Total	51	100,0	100,0	

RVU inicial dicotómica

		Frecuencia	Porcentaje	Porcentaje válido	Porcentaje acumulado
Válido	No	11	21,6	22,0	22,0
	Sí	39	76,5	78,0	100,0
	Total	50	98,0	100,0	
Perdidos	Sistema	1	2,0		
Total		51	100,0		

UHN obstructiva inicial dicotómica

		Frecuencia	Porcentaje	Porcentaje válido	Porcentaje acumulado
Válido	No	30	58,8	61,2	61,2
	Sí	19	37,3	38,8	100,0
	Total	49	96,1	100,0	
Perdidos	Sistema	2	3,9		
Total		51	100,0		

Tratamiento RVU dicotómica

		Frecuencia	Porcentaje	Porcentaje válido	Porcentaje acumulado
Válido	No	36	70,6	72,0	72,0
	Sí	14	27,5	28,0	100,0
	Total	50	98,0	100,0	
Perdidos	Sistema	1	2,0		
Total		51	100,0		

ITUs post Tx recodificada

		Frecuencia	Porcentaje	Porcentaje válido	Porcentaje acumulado
Válido	No	31	60,8	60,8	60,8
	Sí	20	39,2	39,2	100,0
	Total	51	100,0	100,0	

Tratamiento inicial recodificada

		Frecuencia	Porcentaje	Porcentaje válido	Porcentaje acumulado
Válido	Resección	15	29,4	29,4	29,4
	Ureterostomía/vesicostomía	36	70,6	70,6	100,0
	Total	51	100,0	100,0	

Variables cuantitativas - Estadísticos descriptivos

	N	Mínimo	Máximo	Media	Desviación estándar
Tiempo diagnóstico (días)	46	0	2642	132,61	424,917
Tiempo diálisis (meses)	51	0	30	7,08	8,573
Supervivencia injerto (meses)	51	1	299	133,43	83,348
Supervivencia injerto a 10 años (meses)	51	1	120	92,63	40,717
