# Supplementary material for: Long-Term Renal Transplant Outcome in Patients With Posterior Urethral Valves. Prognostic Factors Related to Bladder Dysfunction Management
Source: Front Pediatr. 2021 May 11;9:646923. doi: 10.3389/fped.2021.646923 (PMC8144517; doi:10.3389/fped.2021.646923)

RESULTADOS – ANÁLISIS ESTUDIO TRASPLANTE RENAL EN VUP

Fracaso injerto a 10 años

		Frecuencia	Porcentaje	Porcentaje válido	Porcentaje acumulado
Válido	No	36	70,6	70,6	70,6
	Sí	15	29,4	29,4	100,0
	Total	51	100,0	100,0	

ANÁLISIS BIVARIANTE: primero se calculan asociaciones de las distintas variables con el éxito o fracaso del injerto a 10 años (120 meses).

Ampliación vesical * Fracaso injerto a 10 años – No diferencias significativas

Tabla cruzada

			Fracaso injerto a 10 años		Total
			No	Sí	
Ampliación vesical	No	Recuento	27	13	40
		% dentro de Ampliación vesical	67,5%	32,5%	100,0%
		% dentro de Fracaso injerto a 10 años	75,0%	86,7%	78,4%
		% del total	52,9%	25,5%	78,4%
	Sí	Recuento	9	2	11
		% dentro de Ampliación vesical	81,8%	18,2%	100,0%
		% dentro de Fracaso injerto a 10 años	25,0%	13,3%	21,6%
		% del total	17,6%	3,9%	21,6%
Total		Recuento	36	15	51

Pruebas de chi-cuadrado

	Valor	df	Significación asintótica (bilateral)	Significación exacta (bilateral)	Significación exacta (unilateral)
Chi-cuadrado de Pearson	0,852[a]	1	0,356		
Corrección de continuidad	0,302	1	0,583		
Razón de verosimilitud	0,914	1	0,339		
Prueba exacta de Fisher				0,472	0,300
Asociación lineal por lineal	0,835	1	0,361		
N de casos válidos	51				

a. 1 casillas (25,0%) han esperado un recuento menor que 5. El recuento mínimo esperado es 3,24.

Cateterismo intermitente * Fracaso injerto a 10 años – Existen diferencias entre grupos

Tabla cruzada

			Fracaso injerto a 10 años		
			No	Sí	Total
CI	No	Recuento	23	14	37
		% dentro de CI	62,2%	37,8%	100,0%
		% dentro de Fracaso injerto a 10 años	63,9%	93,3%	72,5%
		% del total	45,1%	27,5%	72,5%
	Sí	Recuento	13	1	14
		% dentro de CI	92,9%	7,1%	100,0%
		% dentro de Fracaso injerto a 10 años	36,1%	6,7%	27,5%
		% del total	25,5%	2,0%	27,5%
Total		Recuento	36	15	51

Pruebas de chi-cuadrado

	Valor	df	Significación asintótica (bilateral)	Significación exacta (bilateral)	Significación exacta (unilateral)
Chi-cuadrado de Pearson	4,609[a]	1	0,032		
Corrección de continuidad	3,249	1	0,071		
Razón de verosimilitud	5,505	1	0,019		
Prueba exacta de Fisher				0,041	0,030
Asociación lineal por lineal	4,519	1	0,034		
N de casos válidos	51				

a. 1 casillas (25,0%) han esperado un recuento menor que 5. El recuento mínimo esperado es 4,12.

<u>Mitrofanoff</u> * Fracaso injerto a 10 años – No diferencias significativas

Tabla cruzada

			Fracaso injerto a 10 años		Total
			No	Sí	
Mitrofanoff	No	Recuento	28	15	43
		% dentro de Mitrofanoff	65,1%	34,9%	100,0%
		% dentro de Fracaso injerto a 10 años	77,8%	100,0%	84,3%
		% del total	54,9%	29,4%	84,3%
	Sí	Recuento	8	0	8
		% dentro de Mitrofanoff	100,0%	0,0%	100,0%
		% dentro de Fracaso injerto a 10 años	22,2%	0,0%	15,7%
		% del total	15,7%	0,0%	15,7%
Total		Recuento	36	15	51

Pruebas de chi-cuadrado

	Valor	df	Significación asintótica (bilateral)	Significación exacta (bilateral)	Significación exacta (unilateral)
Chi-cuadrado de Pearson	3,953[a]	1	0,047		
Corrección de continuidad	2,452	1	0,117		
Razón de verosimilitud	6,173	1	0,013		
Prueba exacta de Fisher				0,087	0,048
Asociación lineal por lineal	3,876	1	0,049		
N de casos válidos	51				

a. 1 casillas (25,0%) han esperado un recuento menor que 5. El recuento mínimo esperado es 2,35.

RVU evol * Fracaso injerto a 10 años – No diferencias significativas

Tabla cruzada

			Fracaso injerto a 10 años		
			No	Sí	Total
RVU evol	No	Recuento	22_a	10_a	32
		% dentro de RVU evol	68,8%	31,3%	100,0%
		% dentro de Fracaso injerto a 10 años	62,9%	76,9%	66,7%
		% del total	45,8%	20,8%	66,7%
	Sí	Recuento	13_a	3_a	16
		% dentro de RVU evol	81,3%	18,8%	100,0%
		% dentro de Fracaso injerto a 10 años	37,1%	23,1%	33,3%
		% del total	27,1%	6,3%	33,3%
Total		Recuento	35	13	48

Pruebas de chi-cuadrado

	Valor	df	Significación asintótica (bilateral)	Significación exacta (bilateral)	Significación exacta (unilateral)
Chi-cuadrado de Pearson	0,844[a]	1	0,358		
Corrección de continuidad	0,330	1	0,566		
Razón de verosimilitud	0,880	1	0,348		
Prueba exacta de Fisher				0,497	0,288
Asociación lineal por lineal	0,826	1	0,363		
N de casos válidos	48				

a. 1 casillas (25,0%) han esperado un recuento menor que 5. El recuento mínimo esperado es 4,33.

RVU al injerto * **Fracaso injerto a 10 años** – No diferencias significativas

Tabla cruzada

			Fracaso injerto a 10 años		
			No	Sí	Total
RVU al injerto	No	Recuento	16	5	21
		% dentro de RVU al injerto	76,2%	23,8%	100,0%
		% dentro de Fracaso injerto a 10 años	48,5%	45,5%	47,7%
		% del total	36,4%	11,4%	47,7%
	Sí	Recuento	17	6	23
		% dentro de RVU al injerto	73,9%	26,1%	100,0%
		% dentro de Fracaso injerto a 10 años	51,5%	54,5%	52,3%
		% del total	38,6%	13,6%	52,3%
Total		Recuento	33	11	44

Pruebas de chi-cuadrado

	Valor	df	Significación asintótica (bilateral)	Significación exacta (bilateral)	Significación exacta (unilateral)
Chi-cuadrado de Pearson	0,030[a]	1	0,862		
Corrección de continuidad	0,000	1	1,000		
Razón de verosimilitud	0,030	1	0,862		
Prueba exacta de Fisher				1,000	0,570
Asociación lineal por lineal	0,030	1	0,863		
N de casos válidos	44				

a. 0 casillas (0,0%) han esperado un recuento menor que 5. El recuento mínimo esperado es 5,25.

<u>Diálisis previa</u> * Fracaso injerto a 10 años – No diferencias significativas

Tabla cruzada

			Fracaso injerto a 10 años		
			No	Sí	Total
Diálisis previa	No	Recuento	13	4	17
		% dentro de Diálisis previa	76,5%	23,5%	100,0%
		% dentro de Fracaso injerto a 10 años	36,1%	26,7%	33,3%
		% del total	25,5%	7,8%	33,3%
	Sí	Recuento	23	11	34
		% dentro de Diálisis previa	67,6%	32,4%	100,0%
		% dentro de Fracaso injerto a 10 años	63,9%	73,3%	66,7%
		% del total	45,1%	21,6%	66,7%
Total		Recuento	36	15	51

Pruebas de chi-cuadrado

	Valor	df	Significación asintótica (bilateral)	Significación exacta (bilateral)	Significación exacta (unilateral)
Chi-cuadrado de Pearson	0,425[a]	1	0,514		
Corrección de continuidad	0,106	1	0,744		
Razón de verosimilitud	0,435	1	0,510		
Prueba exacta de Fisher				0,746	0,378
Asociación lineal por lineal	0,417	1	0,519		
N de casos válidos	51				

a. 0 casillas (0,0%) han esperado un recuento menor que 5. El recuento mínimo esperado es 5,00.

<u>Tipo trasplante</u> * Fracaso injerto a 10 años – Sí existen diferencias entre ambos grupos

Tabla cruzada

			Fracaso injerto a 10 años		
			No	Sí	Total
Tipo trasplante	Cadáver	Recuento	19	13	32
		% dentro de Tipo trasplante	59,4%	40,6%	100,0%
		% dentro de Fracaso injerto a 10 años	52,8%	86,7%	62,7%
		% del total	37,3%	25,5%	62,7%
	Vivo	Recuento	17	2	19
		% dentro de Tipo trasplante	89,5%	10,5%	100,0%
		% dentro de Fracaso injerto a 10 años	47,2%	13,3%	37,3%
		% del total	33,3%	3,9%	37,3%
Total		Recuento	36	15	51

Pruebas de chi-cuadrado

	Valor	df	Significación asintótica (bilateral)	Significación exacta (bilateral)	Significación exacta (unilateral)
Chi-cuadrado de Pearson	5,202[a]	1	0,023		
Corrección de continuidad	3,853	1	0,050		
Razón de verosimilitud	5,775	1	0,016		
Prueba exacta de Fisher				0,028	0,022
Asociación lineal por lineal	5,100	1	0,024		
N de casos válidos	51				

a. 0 casillas (0,0%) han esperado un recuento menor que 5. El recuento mínimo esperado es 5,59.

<u>Tipo inmunosupresión</u> * Fracaso injerto a 10 años – No diferencias significativas

Tabla cruzada

			Fracaso injerto a 10 años		
			No	Sí	Total
Tipo inmunosupresión	Cyc	Recuento	17	9	26
		% dentro de Tipo inmunosupresión	65,4%	34,6%	100,0%
		% dentro de Fracaso injerto a 10 años	47,2%	60,0%	51,0%
		% del total	33,3%	17,6%	51,0%
	Azt	Recuento	19	6	25
		% dentro de Tipo inmunosupresión	76,0%	24,0%	100,0%
		% dentro de Fracaso injerto a 10 años	52,8%	40,0%	49,0%
		% del total	37,3%	11,8%	49,0%
Total		Recuento	36	15	51

Pruebas de chi-cuadrado

	Valor	df	Significación asintótica (bilateral)	Significación exacta (bilateral)	Significación exacta (unilateral)
Chi-cuadrado de Pearson	0,692[a]	1	0,406		
Corrección de continuidad	0,275	1	0,600		
Razón de verosimilitud	0,696	1	0,404		
Prueba exacta de Fisher				0,541	0,301
Asociación lineal por lineal	0,678	1	0,410		
N de casos válidos	51				

a. 0 casillas (0,0%) han esperado un recuento menor que 5. El recuento mínimo esperado es 7,35.

ITUs post-trasplante (recodificada sí/no) * Fracaso injerto a 10 años – Existe tendencia a diferencias significativas

Tabla cruzada

			Fracaso injerto a 10 años		
			No	Sí	Total
ITUs post Tx recodificada	No	Recuento	19	12	31
		% dentro de ITUs post Tx recodificada	61,3%	38,7%	100,0%
		% dentro de Fracaso injerto a 10 años	52,8%	80,0%	60,8%
		% del total	37,3%	23,5%	60,8%
	Sí	Recuento	17	3	20
		% dentro de ITUs post Tx recodificada	85,0%	15,0%	100,0%
		% dentro de Fracaso injerto a 10 años	47,2%	20,0%	39,2%
		% del total	33,3%	5,9%	39,2%
Total		Recuento	36	15	51

Pruebas de chi-cuadrado

	Valor	df	Significación asintótica (bilateral)	Significación exacta (bilateral)	Significación exacta (unilateral)
Chi-cuadrado de Pearson	3,292[a]	1	0,070		
Corrección de continuidad	2,249	1	0,134		
Razón de verosimilitud	3,502	1	0,061		
Prueba exacta de Fisher				0,115	0,065
Asociación lineal por lineal	3,227	1	0,072		
N de casos válidos	51				

a. 0 casillas (0,0%) han esperado un recuento menor que 5. El recuento mínimo esperado es 5,88.

ANÁLISIS DE SUPERVIVENCIA: aportamos curvas de supervivencia y test de log-rank para aquellas variables que muestran diferencias significativas en el tiempo de supervivencia para un máximo de 10 años (120 meses).

<u>Cateterismo intermitente</u> * Supervivencia injerto a 10 años – Existen diferencias significativas en las distribuciones de supervivencia (en meses) entre grupos

MEDIA

CI	Estimación	Error estándar	Intervalo de confianza de 95 % Límite inferior	Límite superior
No	91,119	7,216	76,976	105,263
Sí	113,238	6,516	100,467	126,009
Global	97,171	5,711	85,976	108,365

Comparaciones globales

	Chi-cuadrado	gl	Sig.
Log Rank (Mantel-Cox)	4,266	1	0,039

Prueba de igualdad de distribuciones de supervivencia para los distintos niveles de CI.

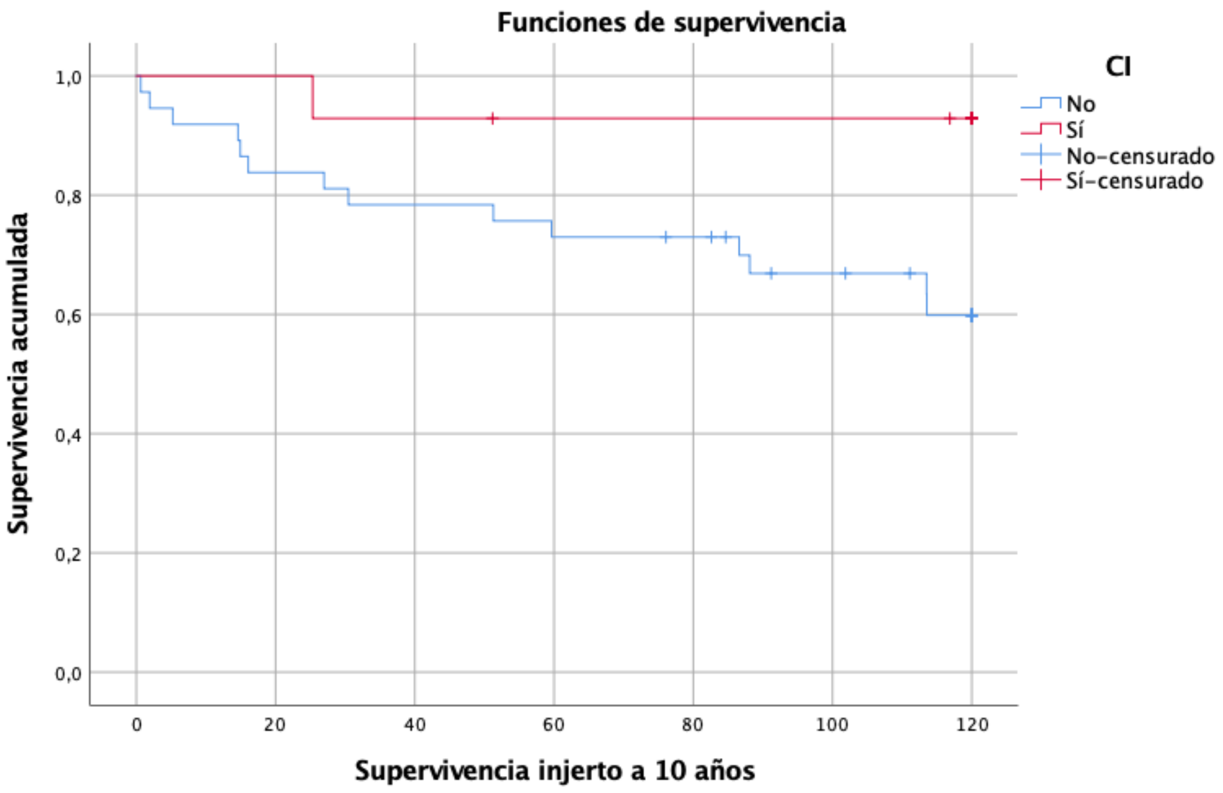

<u>Mitrofanoff</u> * Supervivencia injerto a 10 años – Existen diferencias cuasi significativas en las distribuciones de supervivencia (en meses) entre grupos

***Aquí no se obtienen estadísticos con diferencias en la media del tiempo de supervivencia porque no existen EVENTOS en los pacientes con Mitrofanoff, por lo que el tiempo medio al evento es cero.*

Comparaciones globales

	Chi-cuadrado	gl	Sig.
Log Rank (Mantel-Cox)	3,633	1	0,057

Prueba de igualdad de distribuciones de supervivencia para los distintos niveles de Mitrofanoff.

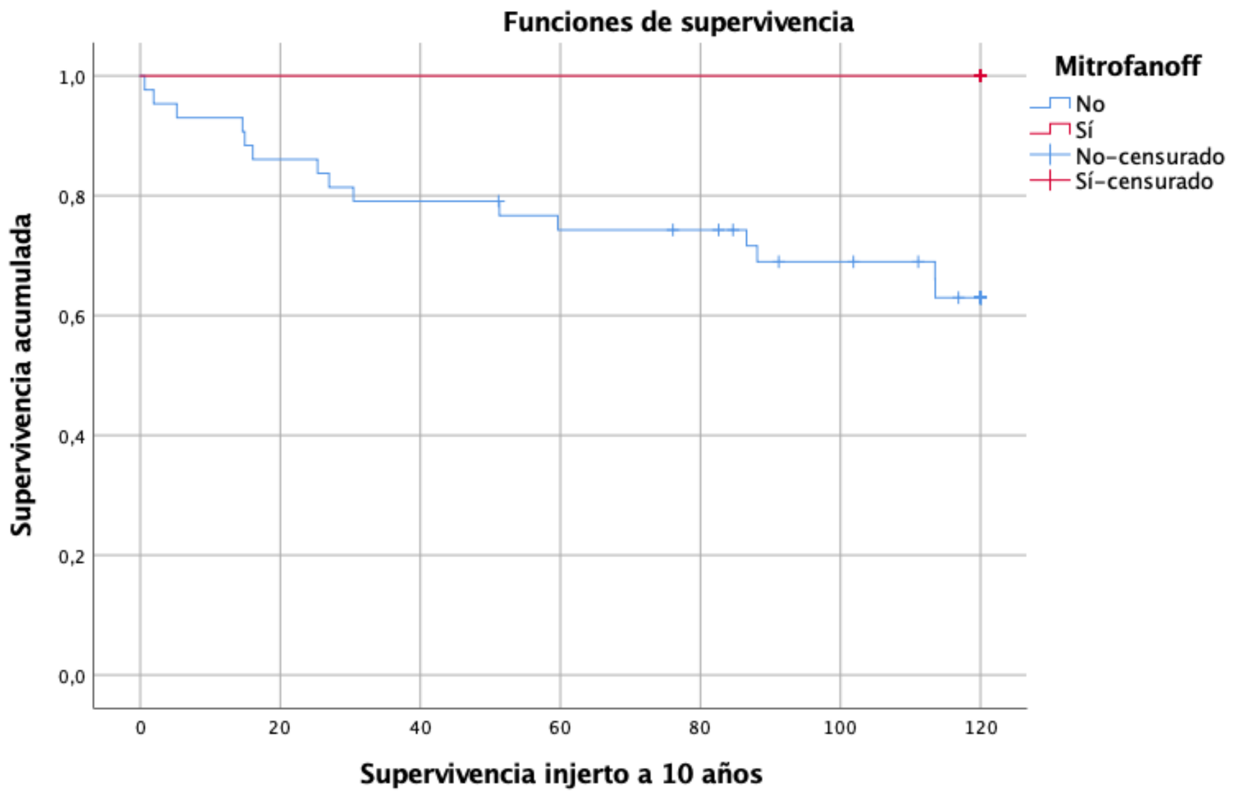

Tipo de trasplante * **Supervivencia injerto a 10 años** – Existen diferencias significativas en las distribuciones de supervivencia (en meses) entre grupos

MEDIA

Tipo trasplante	Estimación	Desv. estándar	Intervalo de confianza de 95 % Límite inferior	Límite superior
Cadáver	87,465	8,017	71,752	103,178
Vivo	114,019	5,319	103,594	124,445
Global	97,171	5,711	85,976	108,365

Comparaciones globales

	Chi-cuadrado	gl	Sig.
Log Rank (Mantel-Cox)	4,442	1	0,035

Prueba de igualdad de distribuciones de supervivencia para los distintos niveles de Tipo trasplante.

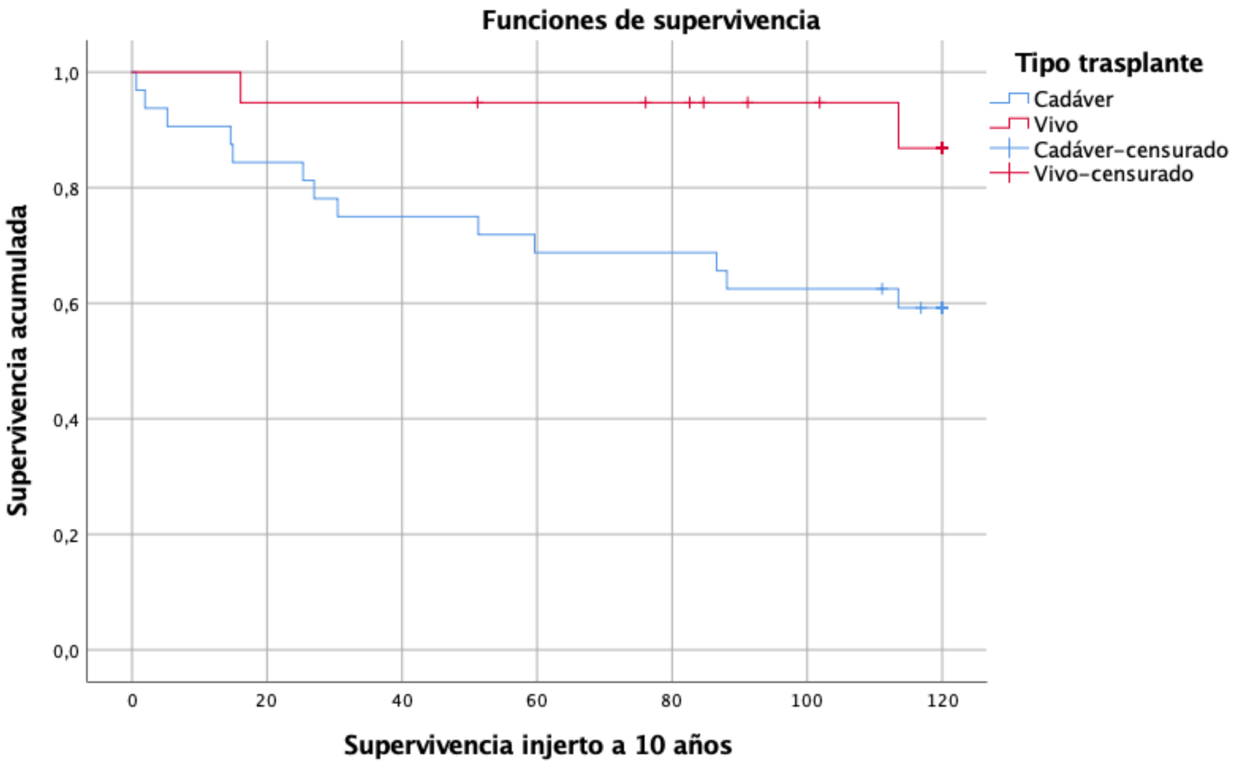

Supplement: Supplementary file 2 [file Data_Sheet_2.PDF]
