# Supplementary material for: Long-Term Renal Transplant Outcome in Patients With Posterior Urethral Valves. Prognostic Factors Related to Bladder Dysfunction Management
Source: Front Pediatr. 2021 May 11;9:646923. doi: 10.3389/fped.2021.646923 (PMC8144517; doi:10.3389/fped.2021.646923)
Supplement: Supplementary file 3 [file Data_Sheet_3.PDF]

COMPARACIÓN DE VARIABLES ENTRE DOS GRUPOS

1. **VARIABLE DE CI**: diferencias entre los grupos de CI sí/no para el resto de las variables estudiadas. Lo ideal aquí es que no existan diferencias para que los grupos sean comparables.

<u>Diagnóstico prenatal</u> * CI – No existen diferencias estadísticamente significativas entre los grupos

			CI		
			No	Sí	Total
Diagnóstico prenatal	No	Recuento	23	9	32
		% dentro de Diagnóstico prenatal	71,9%	28,1%	100,0%
		% dentro de CI	62,2%	64,3%	62,7%
		% del total	45,1%	17,6%	62,7%
	Sí	Recuento	14	5	19
		% dentro de Diagnóstico prenatal	73,7%	26,3%	100,0%
		% dentro de CI	37,8%	35,7%	37,3%
		% del total	27,5%	9,8%	37,3%
Total		Recuento	37	14	51

Pruebas de chi-cuadrado

	Valor	df	Significación asintótica (bilateral)	Significación exacta (bilateral)	Significación exacta (unilateral)
Chi-cuadrado de Pearson	0,020[a]	1	0,889		
Corrección de continuidad	0,000	1	1,000		
Razón de verosimilitud	0,020	1	0,888		
Prueba exacta de Fisher				1,000	0,578
Asociación lineal por lineal	0,019	1	0,890		
N de casos válidos	51				

a. 0 casillas (0,0%) han esperado un recuento menor que 5. El recuento mínimo esperado es 5,22.

Tratamiento intraútero * **CI** – No existen diferencias estadísticamente significativas entre los grupos

Tabla cruzada

			CI		
			No	Sí	Total
Tratamiento intraútero	No	Recuento	36	13	49
		% dentro de Tratamiento intraútero	73,5%	26,5%	100,0%
		% dentro de CI	97,3%	92,9%	96,1%
		% del total	70,6%	25,5%	96,1%
	Ablación	Recuento	1	1	2
		% dentro de Tratamiento intraútero	50,0%	50,0%	100,0%
		% dentro de CI	2,7%	7,1%	3,9%
		% del total	2,0%	2,0%	3,9%
Total		Recuento	37	14	51

Pruebas de chi-cuadrado

	Valor	df	Significación asintótica (bilateral)	Significación exacta (bilateral)	Significación exacta (unilateral)
Chi-cuadrado de Pearson	0,531[a]	1	0,466		
Corrección de continuidad	0,000	1	1,000		
Razón de verosimilitud	0,476	1	0,490		
Prueba exacta de Fisher				0,478	0,478
Asociación lineal por lineal	0,521	1	0,470		
N de casos válidos	51				

a. 2 casillas (50,0%) han esperado un recuento menor que 5. El recuento mínimo esperado es 0,55.

RVU inicial * CI – La prueba de chi-cuadrado NO ES VÁLIDA en este caso por existir más de un 25% de casillas con un recuento menor a 5 y al no ser una tabla 2x2, no se puede aplicar el test exacto de Fisher.

Existen dos alternativas: dicotomizar la variable (RVU sí/no) o dejarla así (en tres grupos: no/unilateral/bilateral) y poner como valor p en la tabla un "NV" o "no válido". Adjunto en la siguiente página la comparación usando la variable dicotomizada para que utilices la opción que prefieras. También sería posible agrupar las categorías de otra manera si existe una forma con más sentido desde la perspectiva clínica. Ocurre lo mismo con otras variables que tienen varios grupos, como UHN obstructiva inicial. Lo señalo en cada caso.

Tabla cruzada

			CI		
			No	Sí	Total
RVU inicial	No	Recuento	9	2	11
		% dentro de RVU inicial	81,8%	18,2%	100,0%
		% dentro de CI	25,0%	14,3%	22,0%
		% del total	18,0%	4,0%	22,0%
	Unilateral	Recuento	6	2	8
		% dentro de RVU inicial	75,0%	25,0%	100,0%
		% dentro de CI	16,7%	14,3%	16,0%
		% del total	12,0%	4,0%	16,0%
	Bilateral	Recuento	21	10	31
		% dentro de RVU inicial	67,7%	32,3%	100,0%
		% dentro de CI	58,3%	71,4%	62,0%
		% del total	42,0%	20,0%	62,0%
Total		Recuento	36	14	50

Pruebas de chi-cuadrado

	Valor	df	Significación asintótica (bilateral)
Chi-cuadrado de Pearson	0,840[a]	2	0,657
Razón de verosimilitud	0,881	2	0,644
Asociación lineal por lineal	0,824	1	0,364
N de casos válidos	50		

a. 2 casillas (33,3%) han esperado un recuento menor que 5. El recuento mínimo esperado es 2,24.

RVU inicial dicotómica * CI – No existen diferencias estadísticamente significativas entre los grupos

Tabla cruzada

			CI No	CI Sí	Total
RVU inicial dicotómica	No	Recuento	9	2	11
		% dentro de RVU inicial dicotómica	81,8%	18,2%	100,0%
		% dentro de CI	25,0%	14,3%	22,0%
		% del total	18,0%	4,0%	22,0%
	Sí	Recuento	27	12	39
		% dentro de RVU inicial dicotómica	69,2%	30,8%	100,0%
		% dentro de CI	75,0%	85,7%	78,0%
		% del total	54,0%	24,0%	78,0%
Total		Recuento	36	14	50

Pruebas de chi-cuadrado

	Valor	df	Significación asintótica (bilateral)	Significación exacta (bilateral)	Significación exacta (unilateral)
Chi-cuadrado de Pearson	0,674[a]	1	0,412		
Corrección de continuidad	0,194	1	0,659		
Razón de verosimilitud	0,719	1	0,396		
Prueba exacta de Fisher				0,705	0,341
Asociación lineal por lineal	0,661	1	0,416		
N de casos válidos	50				

a. 1 casillas (25,0%) han esperado un recuento menor que 5. El recuento mínimo esperado es 3,08.

UHN obstructiva inicial * CI – La prueba de chi-cuadrado NO ES VÁLIDA en este caso por existir más de un 25% de casillas con un recuento menor a 5.

Tabla cruzada

			CI		
			No	Sí	Total
UHN obstructiva inicial	No	Recuento	21	10	31
		% dentro de UHN obstructiva inicial	67,7%	32,3%	100,0%
		% dentro de CI	60,0%	71,4%	63,3%
		% del total	42,9%	20,4%	63,3%
	Unilateral	Recuento	5	2	7
		% dentro de UHN obstructiva inicial	71,4%	28,6%	100,0%
		% dentro de CI	14,3%	14,3%	14,3%
		% del total	10,2%	4,1%	14,3%
	Bilateral	Recuento	9	2	11
		% dentro de UHN obstructiva inicial	81,8%	18,2%	100,0%
		% dentro de CI	25,7%	14,3%	22,4%
		% del total	18,4%	4,1%	22,4%
Total		Recuento	35	14	49

Pruebas de chi-cuadrado

	Valor	df	Significación asintótica (bilateral)
Chi-cuadrado de Pearson	0,788[a]	2	0,674
Razón de verosimilitud	0,838	2	0,658
Asociación lineal por lineal	0,741	1	0,389
N de casos válidos	49		

a. 2 casillas (33,3%) han esperado un recuento menor que 5. El recuento mínimo esperado es 2,00.

UHN obstructiva inicial dicotómica * CI – No existen diferencias estadísticamente significativas entre los grupos

Tabla cruzada

			CI No	CI Sí	Total
UHN obstructiva inicial dicotómica	No	Recuento	20	10	30
		% dentro de UHN obstructiva inicial dicotómica	66,7%	33,3%	100,0%
		% dentro de CI	57,1%	71,4%	61,2%
		% del total	40,8%	20,4%	61,2%
	Sí	Recuento	15	4	19
		% dentro de UHN obstructiva inicial dicotómica	78,9%	21,1%	100,0%
		% dentro de CI	42,9%	28,6%	38,8%
		% del total	30,6%	8,2%	38,8%
Total		Recuento	35	14	49

Pruebas de chi-cuadrado

	Valor	df	Significación asintótica (bilateral)	Significación exacta (bilateral)	Significación exacta (unilateral)
Chi-cuadrado de Pearson	0,860[a]	1	0,354		
Corrección de continuidad	0,363	1	0,547		
Razón de verosimilitud	0,883	1	0,347		
Prueba exacta de Fisher				0,518	0,276
Asociación lineal por lineal	0,842	1	0,359		
N de casos válidos	49				

a. 0 casillas (0,0%) han esperado un recuento menor que 5. El recuento mínimo esperado es 5,43.

Tratamiento inicial *** CI** – La prueba de chi-cuadrado NO ES VÁLIDA en este caso por existir más de un 25% de casillas con un recuento menor a 5.

Tabla cruzada

			CI		
			No	Sí	Total
Tratamiento inicial	Resección	Recuento	12	3	15
		% dentro de Tratamiento inicial	80,0%	20,0%	100,0%
		% dentro de CI	32,4%	21,4%	29,4%
		% del total	23,5%	5,9%	29,4%
	Ureterostomía	Recuento	23	10	33
		% dentro de Tratamiento inicial	69,7%	30,3%	100,0%
		% dentro de CI	62,2%	71,4%	64,7%
		% del total	45,1%	19,6%	64,7%
	Vesicostomía	Recuento	2	1	3
		% dentro de Tratamiento inicial	66,7%	33,3%	100,0%
		% dentro de CI	5,4%	7,1%	5,9%
		% del total	3,9%	2,0%	5,9%
Total		Recuento	37	14	51

Pruebas de chi-cuadrado

	Valor	df	Significación asintótica (bilateral)
Chi-cuadrado de Pearson	0,605[a]	2	0,739
Razón de verosimilitud	0,628	2	0,730
Asociación lineal por lineal	0,543	1	0,461
N de casos válidos	51		

a. 3 casillas (50,0%) han esperado un recuento menor que 5. El recuento mínimo esperado es ,82.

Tratamiento inicial dicotómica * CI – No existen diferencias estadísticamente significativas entre los grupos

Tabla cruzada

			CI		
			No	Sí	Total
Tratamiento inicial recodificada	Resección	Recuento	12	3	15
		% dentro de Tratamiento inicial recodificada	80,0%	20,0%	100,0%
		% dentro de CI	32,4%	21,4%	29,4%
		% del total	23,5%	5,9%	29,4%
	Ureterostomía/ vesicostomía	Recuento	25	11	36
		% dentro de Tratamiento inicial recodificada	69,4%	30,6%	100,0%
		% dentro de CI	67,6%	78,6%	70,6%
		% del total	49,0%	21,6%	70,6%
Total		Recuento	37	14	51

Pruebas de chi-cuadrado

	Valor	df	Significación asintótica (bilateral)	Significación exacta (bilateral)	Significación exacta (unilateral)
Chi-cuadrado de Pearson	0,592[a]	1	0,442		
Corrección de continuidad	0,181	1	0,671		
Razón de verosimilitud	0,617	1	0,432		
Prueba exacta de Fisher				0,513	0,343
Asociación lineal por lineal	0,581	1	0,446		
N de casos válidos	51				

a. 1 casillas (25,0%) han esperado un recuento menor que 5. El recuento mínimo esperado es 4,12.

<u>Nefrectomía</u> * CI – No existen diferencias estadísticamente significativas entre los grupos

Tabla cruzada

			CI		
			No	Sí	Total
Nefrectomía	No	Recuento	10	1	11
		% dentro de Nefrectomía	90,9%	9,1%	100,0%
		% dentro de CI	27,8%	7,1%	22,0%
		% del total	20,0%	2,0%	22,0%
	Unilateral	Recuento	11	7	18
		% dentro de Nefrectomía	61,1%	38,9%	100,0%
		% dentro de CI	30,6%	50,0%	36,0%
		% del total	22,0%	14,0%	36,0%
	Bilateral	Recuento	15	6	21
		% dentro de Nefrectomía	71,4%	28,6%	100,0%
		% dentro de CI	41,7%	42,9%	42,0%
		% del total	30,0%	12,0%	42,0%
Total		Recuento	36	14	50

Cada letra del subíndice denota un subconjunto de CI categorías cuyas proporciones de columna no difieren de forma significativa entre sí en el nivel ,05.

Pruebas de chi-cuadrado

	Valor	df	Significación asintótica (bilateral)
Chi-cuadrado de Pearson	3,013[a]	2	0,222
Razón de verosimilitud	3,409	2	0,182
Asociación lineal por lineal	0,784	1	0,376
N de casos válidos	50		

a. 1 casillas (16,7%) han esperado un recuento menor que 5. El recuento mínimo esperado es 3,08.

Ampliación vesical * **CI** – Sí existen diferencias significativas entre los grupos. Podría actuar como factor de confusión

Tabla cruzada

			CI		
			No	Sí	Total
Ampliación vesical	No	Recuento	36	4	40
		% dentro de Ampliación vesical	90,0%	10,0%	100,0%
		% dentro de CI	97,3%	28,6%	78,4%
		% del total	70,6%	7,8%	78,4%
	Sí	Recuento	1	10	11
		% dentro de Ampliación vesical	9,1%	90,9%	100,0%
		% dentro de CI	2,7%	71,4%	21,6%
		% del total	2,0%	19,6%	21,6%
Total		Recuento	37	14	51

Pruebas de chi-cuadrado

	Valor	df	Significación asintótica (bilateral)	Significación exacta (bilateral)	Significación exacta (unilateral)
Chi-cuadrado de Pearson	28,359[a]	1	0,000		
Corrección de continuidad	24,442	1	0,000		
Razón de verosimilitud	27,236	1	0,000		
Prueba exacta de Fisher				0,000	0,000
Asociación lineal por lineal	27,803	1	0,000		
N de casos válidos	51				

a. 1 casillas (25,0%) han esperado un recuento menor que 5. El recuento mínimo esperado es 3,02.

***No hago la comparación con la variable de "tipo de ampliación" porque en este caso, aunque se agrupe, sigue sin ser posible obtener una prueba válida.*

<u>Anticolinérgicos</u> * CI – Sí existen diferencias estadísticamente significativas entre los grupos. Podría actuar como factor de confusión

Tabla cruzada

			CI		
			No	Sí	Total
Anticolinérgicos	No	Recuento	35	7	42
		% dentro de Anticolinérgicos	83,3%	16,7%	100,0%
		% dentro de CI	94,6%	50,0%	82,4%
		% del total	68,6%	13,7%	82,4%
	Sí	Recuento	2	7	9
		% dentro de Anticolinérgicos	22,2%	77,8%	100,0%
		% dentro de CI	5,4%	50,0%	17,6%
		% del total	3,9%	13,7%	17,6%
Total		Recuento	37	14	51

Pruebas de chi-cuadrado

	Valor	df	Significación asintótica (bilateral)	Significación exacta (bilateral)	Significación exacta (unilateral)
Chi-cuadrado de Pearson	13,899[a]	1	0,000		
Corrección de continuidad	10,999	1	0,001		
Razón de verosimilitud	12,563	1	0,000		
Prueba exacta de Fisher				0,001	0,001
Asociación lineal por lineal	13,626	1	0,000		
N de casos válidos	51				

a. 1 casillas (25,0%) han esperado un recuento menor que 5. El recuento mínimo esperado es 2,47.

Mitrofanoff * **CI** – Sí existen diferencias estadísticamente significativas entre los grupos. Podría actuar como factor de confusión

Tabla cruzada

			CI		
			No	Sí	Total
Mitrofanoff	No	Recuento	37	6	43
		% dentro de Mitrofanoff	86,0%	14,0%	100,0%
		% dentro de CI	100,0%	42,9%	84,3%
		% del total	72,5%	11,8%	84,3%
	Sí	Recuento	0	8	8
		% dentro de Mitrofanoff	0,0%	100,0%	100,0%
		% dentro de CI	0,0%	57,1%	15,7%
		% del total	0,0%	15,7%	15,7%
Total		Recuento	37	14	51

Pruebas de chi-cuadrado

	Valor	df	Significación asintótica (bilateral)	Significación exacta (bilateral)	Significación exacta (unilateral)
Chi-cuadrado de Pearson	25,076[a]	1	0,000		
Corrección de continuidad	20,942	1	0,000		
Razón de verosimilitud	25,191	1	0,000		
Prueba exacta de Fisher				0,000	0,000
Asociación lineal por lineal	24,585	1	0,000		
N de casos válidos	51				

a. 1 casillas (25,0%) han esperado un recuento menor que 5. El recuento mínimo esperado es 2,20.

***No hago la comparación con la variable de "tipo Mitrofanoff" porque en este caso, aunque se agrupe, sigue sin ser posible obtener una prueba válida.*

<u>RVU evol</u> * CI – No existen diferencias estadísticamente significativas entre los grupos

Tabla cruzada

			CI		
			No	Sí	Total
RVU evol	No	Recuento	23	9	32
		% dentro de RVU evol	71,9%	28,1%	100,0%
		% dentro de CI	67,6%	64,3%	66,7%
		% del total	47,9%	18,8%	66,7%
	Sí	Recuento	11	5	16
		% dentro de RVU evol	68,8%	31,3%	100,0%
		% dentro de CI	32,4%	35,7%	33,3%
		% del total	22,9%	10,4%	33,3%
Total		Recuento	34	14	48

Pruebas de chi-cuadrado

	Valor	df	Significación asintótica (bilateral)	Significación exacta (bilateral)	Significación exacta (unilateral)
Chi-cuadrado de Pearson	0,050[a]	1	0,822		
Corrección de continuidad[b]	0,000	1	1,000		
Razón de verosimilitud	0,050	1	0,823		
Prueba exacta de Fisher				1,000	0,538
Asociación lineal por lineal	0,049	1	0,824		
N de casos válidos	48				

a. 1 casillas (25,0%) han esperado un recuento menor que 5. El recuento mínimo esperado es 4,67.

***No hago la comparación con la variable de “grado RVU” ni con “tratamiento RVU” porque la prueba de chi-cuadrado no es válida y no veo posibilidad de dicotomizarlas correctamente.*

<u>RVU al injerto</u> * CI – No existen diferencias estadísticamente significativas entre los grupos

Tabla cruzada

			CI		
			No	Sí	Total
RVU al injerto	No	Recuento	15	6	21
		% dentro de RVU al injerto	71,4%	28,6%	100,0%
		% dentro de CI	48,4%	46,2%	47,7%
		% del total	34,1%	13,6%	47,7%
	Sí	Recuento	16	7	23
		% dentro de RVU al injerto	69,6%	30,4%	100,0%
		% dentro de CI	51,6%	53,8%	52,3%
		% del total	36,4%	15,9%	52,3%
Total		Recuento	31	13	44

Pruebas de chi-cuadrado

	Valor	df	Significación asintótica (bilateral)	Significación exacta (bilateral)	Significación exacta (unilateral)
Chi-cuadrado de Pearson	0,018[a]	1	0,892		
Corrección de continuidad	0,000	1	1,000		
Razón de verosimilitud	0,018	1	0,892		
Prueba exacta de Fisher				1,000	0,578
Asociación lineal por lineal	0,018	1	0,894		
N de casos válidos	44				

a. 0 casillas (,0%) han esperado un recuento menor que 5. El recuento mínimo esperado es 6,20.

***No hago la comparación con la variable de "grado RVU injerto" porque la prueba de chi-cuadrado no es válida y no veo posibilidad de dicotomizarla correctamente.*

<u>Diálisis previa</u> * CI – No existen diferencias estadísticamente significativas entre los grupos

Tabla cruzada

			CI		
			No	Sí	Total
Diálisis previa	No	Recuento	11	6	17
		% dentro de Diálisis previa	64,7%	35,3%	100,0%
		% dentro de CI	29,7%	42,9%	33,3%
		% del total	21,6%	11,8%	33,3%
	Sí	Recuento	26	8	34
		% dentro de Diálisis previa	76,5%	23,5%	100,0%
		% dentro de CI	70,3%	57,1%	66,7%
		% del total	51,0%	15,7%	66,7%
Total		Recuento	37	14	51

Pruebas de chi-cuadrado

	Valor	df	Significación asintótica (bilateral)	Significación exacta (bilateral)	Significación exacta (unilateral)
Chi-cuadrado de Pearson	0,788[a]	1	0,375		
Corrección de continuidad[b]	0,308	1	0,579		
Razón de verosimilitud	0,770	1	0,380		
Prueba exacta de Fisher				0,508	0,286
Asociación lineal por lineal	0,772	1	0,380		
N de casos válidos	51				

a. 1 casillas (25,0%) han esperado un recuento menor que 5. El recuento mínimo esperado es 4,67.

***No hago la comparación con la variable de "tipo diálisis" porque la prueba de chi-cuadrado no es válida y no veo posibilidad de dicotomizarla correctamente.*

Tipo trasplante * CI – No existen diferencias estadísticamente significativas entre los grupos

Tabla cruzada

			CI		
			No	Sí	Total
Tipo trasplante	Cadáver	Recuento	23	9	32
		% dentro de Tipo trasplante	71,9%	28,1%	100,0%
		% dentro de CI	62,2%	64,3%	62,7%
		% del total	45,1%	17,6%	62,7%
	Vivo	Recuento	14	5	19
		% dentro de Tipo trasplante	73,7%	26,3%	100,0%
		% dentro de CI	37,8%	35,7%	37,3%
		% del total	27,5%	9,8%	37,3%
Total		Recuento	37	14	51

Pruebas de chi-cuadrado

	Valor	df	Significación asintótica (bilateral)	Significación exacta (bilateral)	Significación exacta (unilateral)
Chi-cuadrado de Pearson	0,020[a]	1	0,889		
Corrección de continuidad	0,000	1	1,000		
Razón de verosimilitud	0,020	1	0,888		
Prueba exacta de Fisher				1,000	0,578
Asociación lineal por lineal	0,019	1	0,890		
N de casos válidos	51				

a. 0 casillas (,0%) han esperado un recuento menor que 5. El recuento mínimo esperado es 5,22.

<u>Número de rechazos agudos</u> * CI – La prueba de chi-cuadrado NO ES VÁLIDA en este caso por existir más de un 25% de casillas con un recuento menor a 5

Tabla cruzada

			CI		
			No	Sí	Total
Número rechazos agudos	0	Recuento	27	12	39
		% dentro de Número rechazos agudos	69,2%	30,8%	100,0%
		% dentro de CI	73,0%	85,7%	76,5%
		% del total	52,9%	23,5%	76,5%
	1	Recuento	8	2	10
		% dentro de Número rechazos agudos	80,0%	20,0%	100,0%
		% dentro de CI	21,6%	14,3%	19,6%
		% del total	15,7%	3,9%	19,6%
	3	Recuento	1	0	1
		% dentro de Número rechazos agudos	100,0%	0,0%	100,0%
		% dentro de CI	2,7%	0,0%	2,0%
		% del total	2,0%	0,0%	2,0%
	4	Recuento	1	0	1
		% dentro de Número rechazos agudos	100,0%	0,0%	100,0%
		% dentro de CI	2,7%	0,0%	2,0%
		% del total	2,0%	0,0%	2,0%
Total		Recuento	37	14	51

Pruebas de chi-cuadrado

	Valor	df	Significación asintótica (bilateral)
Chi-cuadrado de Pearson	1,251[a]	3	0,741
Razón de verosimilitud	1,792	3	0,617
Asociación lineal por lineal	1,193	1	0,275
N de casos válidos	51		

a. 5 casillas (62,5%) han esperado un recuento menor que 5. El recuento mínimo esperado es ,27.

***Aquí se podría dicotomizar entre rechazos agudos sí/no, pero no sé si es relevante para vosotros. Avísame si quieres que lo haga.*

Complicaciones urológicas * **CI** – No existen diferencias estadísticamente significativas entre los grupos

Tabla cruzada

			CI		
			No	Sí	Total
Complicaciones urológicas	No	Recuento	36	14	50
		% dentro de Complicaciones urológicas	72,0%	28,0%	100,0%
		% dentro de CI	97,3%	100,0%	98,0%
		% del total	70,6%	27,5%	98,0%
	Sí	Recuento	1	0	1
		% dentro de Complicaciones urológicas	100,0%	0,0%	100,0%
		% dentro de CI	2,7%	0,0%	2,0%
		% del total	2,0%	0,0%	2,0%
Total		Recuento	37	14	51

Pruebas de chi-cuadrado

	Valor	df	Significación asintótica (bilateral)	Significación exacta (bilateral)	Significación exacta (unilateral)
Chi-cuadrado de Pearson	0,386[a]	1	0,534		
Corrección de continuidad	0,000	1	1,000		
Razón de verosimilitud	0,649	1	0,420		
Prueba exacta de Fisher				1,000	0,725
Asociación lineal por lineal	0,378	1	0,538		
N de casos válidos	51				

a. 2 casillas (50,0%) han esperado un recuento menor que 5. El recuento mínimo esperado es ,27.

Complicaciones vasculares * **CI** – No existen diferencias estadísticamente significativas entre los grupos

Tabla cruzada

			CI		
			No	Sí	Total
Complicaciones vasculares	No	Recuento	36	13	49
		% dentro de Complicaciones vasculares	73,5%	26,5%	100,0%
		% dentro de CI	97,3%	92,9%	96,1%
		% del total	70,6%	25,5%	96,1%
	Sí	Recuento	1	1	2
		% dentro de Complicaciones vasculares	50,0%	50,0%	100,0%
		% dentro de CI	2,7%	7,1%	3,9%
		% del total	2,0%	2,0%	3,9%
Total		Recuento	37	14	51

Pruebas de chi-cuadrado

	Valor	df	Significación asintótica (bilateral)	Significación exacta (bilateral)	Significación exacta (unilateral)
Chi-cuadrado de Pearson	0,531[a]	1	0,466		
Corrección de continuidad	0,000	1	1,000		
Razón de verosimilitud	0,476	1	0,490		
Prueba exacta de Fisher				0,478	0,478
Asociación lineal por lineal	0,521	1	0,470		
N de casos válidos	51				

a. 2 casillas (50,0%) han esperado un recuento menor que 5. El recuento mínimo esperado es ,55.

Tipo inmunosupresión * CI – No existen diferencias estadísticamente significativas entre los grupos

Tabla cruzada

			CI		
			No	Sí	Total
Tipo inmunosupresión	Cyc	Recuento	18	8	26
		% dentro de Tipo inmunosupresión	69,2%	30,8%	100,0%
		% dentro de CI	48,6%	57,1%	51,0%
		% del total	35,3%	15,7%	51,0%
	Azt	Recuento	19	6	25
		% dentro de Tipo inmunosupresión	76,0%	24,0%	100,0%
		% dentro de CI	51,4%	42,9%	49,0%
		% del total	37,3%	11,8%	49,0%
Total		Recuento	37	14	51

Pruebas de chi-cuadrado

	Valor	df	Significación asintótica (bilateral)	Significación exacta (bilateral)	Significación exacta (unilateral)
Chi-cuadrado de Pearson	0,293[a]	1	0,588		
Corrección de continuidad	0,052	1	0,820		
Razón de verosimilitud	0,294	1	0,588		
Prueba exacta de Fisher				0,755	0,411
Asociación lineal por lineal	0,287	1	0,592		
N de casos válidos	51				

a. 0 casillas (,0%) han esperado un recuento menor que 5. El recuento mínimo esperado es 6,86.

<u>ITUs de repetición</u> * CI – Sí existen diferencias estadísticamente significativas entre los grupos. Podría actuar como factor de confusión

Tabla cruzada

			CI		
			No	Sí	Total
ITUs de repetición	No	Recuento	33	2	35
		% dentro de ITUs de repetición	94,3%	5,7%	100,0%
		% dentro de CI	89,2%	14,3%	68,6%
		% del total	64,7%	3,9%	68,6%
	Sí	Recuento	4	12	16
		% dentro de ITUs de repetición	25,0%	75,0%	100,0%
		% dentro de CI	10,8%	85,7%	31,4%
		% del total	7,8%	23,5%	31,4%
Total		Recuento	37	14	51

Pruebas de chi-cuadrado

	Valor	df	Significación asintótica (bilateral)	Significación exacta (bilateral)	Significación exacta (unilateral)
Chi-cuadrado de Pearson	26,468[a]	1	0,000		
Corrección de continuidad	23,103	1	0,000		
Razón de verosimilitud	26,618	1	0,000		
Prueba exacta de Fisher				0,000	0,000
Asociación lineal por lineal	25,949	1	0,000		
N de casos válidos	51				

a. 1 casillas (25,0%) han esperado un recuento menor que 5. El recuento mínimo esperado es 4,39.

Edad al trasplante (en años) * CI - No existen diferencias entre los grupos. Prueba de la t de Student con un nivel de significación de 0,837.

	CI	N	Media	Desv. estándar
Edad al trasplante (años)	No	37	6,9212	4,89524
	Sí	14	7,2381	4,82580
