# Supplementary material for: Long-Term Renal Transplant Outcome in Patients With Posterior Urethral Valves. Prognostic Factors Related to Bladder Dysfunction Management
Source: Front Pediatr. 2021 May 11;9:646923. doi: 10.3389/fped.2021.646923 (PMC8144517; doi:10.3389/fped.2021.646923)
Supplement: Supplementary file 4 [file Data_Sheet_4.PDF]

COMPARACIÓN DE VARIABLES ENTRE DOS GRUPOS

1. **VARIABLE DE MITROFANOFF**: diferencias entre los grupos de Mitrofanoff sí/no para el resto de las variables estudiadas. Lo ideal aquí es que no existan diferencias para que los grupos sean comparables.

Diagnóstico prenatal * Mitrofanoff – No existen diferencias estadísticamente significativas entre los grupos

Tabla cruzada

			Mitrofanoff		
			No	Sí	Total
Diagnóstico prenatal	No	Recuento	27	5	32
		% dentro de Diagnóstico prenatal	84,4%	15,6%	100,0%
		% dentro de Mitrofanoff	62,8%	62,5%	62,7%
		% del total	52,9%	9,8%	62,7%
	Sí	Recuento	16	3	19
		% dentro de Diagnóstico prenatal	84,2%	15,8%	100,0%
		% dentro de Mitrofanoff	37,2%	37,5%	37,3%
		% del total	31,4%	5,9%	37,3%
Total		Recuento	43	8	51

Pruebas de chi-cuadrado

	Valor	df	Significación asintótica (bilateral)	Significación exacta (bilateral)	Significación exacta (unilateral)
Chi-cuadrado de Pearson	0,000[a]	1	0,988		
Corrección de continuidad	0,000	1	1,000		
Razón de verosimilitud	0,000	1	0,988		
Prueba exacta de Fisher				1,000	0,640
Asociación lineal por lineal	0,000	1	0,988		
N de casos válidos	51				

a. 1 casillas (25,0%) han esperado un recuento menor que 5. El recuento mínimo esperado es 2,98.

Tratamiento intraútero * **Mitrofanoff** – No existen diferencias estadísticamente significativas entre los grupos

Tabla cruzada

			Mitrofanoff No	Mitrofanoff Sí	Total
Tratamiento intraútero	No	Recuento	41	8	49
		% dentro de Tratamiento intraútero	83,7%	16,3%	100,0%
		% dentro de Mitrofanoff	95,3%	100,0%	96,1%
		% del total	80,4%	15,7%	96,1%
	Ablación	Recuento	2	0	2
		% dentro de Tratamiento intraútero	100,0%	0,0%	100,0%
		% dentro de Mitrofanoff	4,7%	0,0%	3,9%
		% del total	3,9%	0,0%	3,9%
Total		Recuento	43	8	51

Pruebas de chi-cuadrado

	Valor	df	Significación asintótica (bilateral)	Significación exacta (bilateral)	Significación exacta (unilateral)
Chi-cuadrado de Pearson	0,387[a]	1	0,534		
Corrección de continuidad	0,000	1	1,000		
Razón de verosimilitud	0,698	1	0,404		
Prueba exacta de Fisher				1,000	0,708
Asociación lineal por lineal	0,380	1	0,538		
N de casos válidos	51				

a. 2 casillas (50,0%) han esperado un recuento menor que 5. El recuento mínimo esperado es 0,31.

RVU inicial * Mitrofanoff – La prueba de chi-cuadrado NO ES VÁLIDA en este caso por existir más de un 25% de casillas con un recuento menor a 5 y al no ser una tabla 2x2, no se puede aplicar el test exacto de Fisher.

Existen dos alternativas: dicotomizar la variable (RVU sí/no) o dejarla así (en tres grupos: no/unilateral/bilateral) y poner como valor p en la tabla un "NV" o "no válido". Adjunto en la siguiente página la comparación usando la variable dicotomizada para que utilices la opción que prefieras. También sería posible agrupar las categorías de otra manera si existe una forma con más sentido desde la perspectiva clínica. Ocurre lo mismo con otras variables que tienen varios grupos, como UHN obstructiva inicial. Lo señalo en cada caso.

Tabla cruzada

			Mitrofanoff		
			No	Sí	Total
RVU inicial	No	Recuento	11	0	11
		% dentro de RVU inicial	100,0%	0,0%	100,0%
		% dentro de Mitrofanoff	26,2%	0,0%	22,0%
		% del total	22,0%	0,0%	22,0%
	Unilateral	Recuento	7	1	8
		% dentro de RVU inicial	87,5%	12,5%	100,0%
		% dentro de Mitrofanoff	16,7%	12,5%	16,0%
		% del total	14,0%	2,0%	16,0%
	Bilateral	Recuento	24	7	31
		% dentro de RVU inicial	77,4%	22,6%	100,0%
		% dentro de Mitrofanoff	57,1%	87,5%	62,0%
		% del total	48,0%	14,0%	62,0%
Total		Recuento	42	8	50

Pruebas de chi-cuadrado

	Valor	df	Significación asintótica (bilateral)
Chi-cuadrado de Pearson	3,167[a]	2	0,205
Razón de verosimilitud	4,821	2	0,090
Asociación lineal por lineal	3,097	1	0,078
N de casos válidos	50		

a. 3 casillas (50,0%) han esperado un recuento menor que 5. El recuento mínimo esperado es 1,28.

RVU inicial dicotómica * Mitrofanoff – No existen diferencias estadísticamente significativas entre los grupos

Tabla cruzada

			Mitrofanoff No	Mitrofanoff Sí	Total
RVU inicial dicotómica	No	Recuento	11	0	11
		% dentro de RVU inicial dicotómica	100,0%	0,0%	100,0%
		% dentro de Mitrofanoff	26,2%	0,0%	22,0%
		% del total	22,0%	0,0%	22,0%
	Sí	Recuento	31	8	39
		% dentro de RVU inicial dicotómica	79,5%	20,5%	100,0%
		% dentro de Mitrofanoff	73,8%	100,0%	78,0%
		% del total	62,0%	16,0%	78,0%
Total		Recuento	42	8	50

Pruebas de chi-cuadrado

	Valor	df	Significación asintótica (bilateral)	Significación exacta (bilateral)	Significación exacta (unilateral)
Chi-cuadrado de Pearson	2,686[a]	1	0,101		
Corrección de continuidad	1,377	1	0,241		
Razón de verosimilitud	4,387	1	0,036		
Prueba exacta de Fisher				0,174	0,115
Asociación lineal por lineal	2,632	1	0,105		
N de casos válidos	50				

a. 1 casillas (25,0%) han esperado un recuento menor que 5. El recuento mínimo esperado es 1,76.

UHN obstructiva inicial * **Mitrofanoff** – La prueba de chi-cuadrado NO ES VÁLIDA en este caso por existir más de un 25% de casillas con un recuento menor a 5.

Tabla cruzada

			Mitrofanoff		
			No	Sí	Total
UHN obstructiva inicial	No	Recuento	24	7	31
		% dentro de UHN obstructiva inicial	77,4%	22,6%	100,0%
		% dentro de Mitrofanoff	58,5%	87,5%	63,3%
		% del total	49,0%	14,3%	63,3%
	Unilateral	Recuento	6	1	7
		% dentro de UHN obstructiva inicial	85,7%	14,3%	100,0%
		% dentro de Mitrofanoff	14,6%	12,5%	14,3%
		% del total	12,2%	2,0%	14,3%
	Bilateral	Recuento	11	0	11
		% dentro de UHN obstructiva inicial	100,0%	0,0%	100,0%
		% dentro de Mitrofanoff	26,8%	0,0%	22,4%
		% del total	22,4%	0,0%	22,4%
Total		Recuento	41	8	49

Pruebas de chi-cuadrado

	Valor	df	Significación asintótica (bilateral)
Chi-cuadrado de Pearson	3,055[a]	2	0,217
Razón de verosimilitud	4,755	2	0,093
Asociación lineal por lineal	2,956	1	0,086
N de casos válidos	49		

a. 2 casillas (33,3%) han esperado un recuento menor que 5. El recuento mínimo esperado es 1,14.

UHN obstructiva inicial dicotómica * Mitrofanoff – No existen diferencias estadísticamente significativas entre los grupos

Tabla cruzada

			Mitrofanoff No	Mitrofanoff Sí	Total
UHN obstructiva inicial dicotómica	No	Recuento	23	7	30
		% dentro de UHN obstructiva inicial dicotómica	76,7%	23,3%	100,0%
		% dentro de Mitrofanoff	56,1%	87,5%	61,2%
		% del total	46,9%	14,3%	61,2%
	Sí	Recuento	18	1	19
		% dentro de UHN obstructiva inicial dicotómica	94,7%	5,3%	100,0%
		% dentro de Mitrofanoff	43,9%	12,5%	38,8%
		% del total	36,7%	2,0%	38,8%
Total		Recuento	41	8	49

Pruebas de chi-cuadrado

	Valor	df	Significación asintótica (bilateral)	Significación exacta (bilateral)	Significación exacta (unilateral)
Chi-cuadrado de Pearson	2,780[a]	1	0,095		
Corrección de continuidad	1,615	1	0,204		
Razón de verosimilitud	3,183	1	0,074		
Prueba exacta de Fisher				0,128	0,099
Asociación lineal por lineal	2,724	1	0,099		
N de casos válidos	49				

a. 2 casillas (50,0%) han esperado un recuento menor que 5. El recuento mínimo esperado es 3,10.

Tratamiento inicial * **Mitrofanoff** – La prueba de chi-cuadrado NO ES VÁLIDA

Tabla cruzada

			Mitrofanoff		
			No	Sí	Total
Tratamiento inicial	Resección	Recuento	13	2	15
		% dentro de Tratamiento inicial	86,7%	13,3%	100,0%
		% dentro de Mitrofanoff	30,2%	25,0%	29,4%
		% del total	25,5%	3,9%	29,4%
	Ureterostomía	Recuento	28	5	33
		% dentro de Tratamiento inicial	84,8%	15,2%	100,0%
		% dentro de Mitrofanoff	65,1%	62,5%	64,7%
		% del total	54,9%	9,8%	64,7%
	Vesicostomía	Recuento	2	1	3
		% dentro de Tratamiento inicial	66,7%	33,3%	100,0%
		% dentro de Mitrofanoff	4,7%	12,5%	5,9%
		% del total	3,9%	2,0%	5,9%
Total		Recuento	43	8	51

Pruebas de chi-cuadrado

	Valor	df	Significación asintótica (bilateral)
Chi-cuadrado de Pearson	0,776[a]	2	0,678
Razón de verosimilitud	0,641	2	0,726
Asociación lineal por lineal	0,380	1	0,537
N de casos válidos	51		

a. 3 casillas (50,0%) han esperado un recuento menor que 5. El recuento mínimo esperado es 0,47.

Tratamiento inicial dicotómica * Mitrofanoff – No existen diferencias estadísticamente significativas entre los grupos

Tabla cruzada

			Mitrofanoff		
			No	Sí	Total
Tratamiento inicial recodificada	Resección	Recuento	13	2	15
		% dentro de Tratamiento inicial recodificada	86,7%	13,3%	100,0%
		% dentro de Mitrofanoff	30,2%	25,0%	29,4%
		% del total	25,5%	3,9%	29,4%
	Ureterostomía/ vesicostomía	Recuento	30	6	36
		% dentro de Tratamiento inicial recodificada	83,3%	16,7%	100,0%
		% dentro de Mitrofanoff	69,8%	75,0%	70,6%
		% del total	58,8%	11,8%	70,6%
Total		Recuento	43	8	51
		% dentro de Tratamiento inicial recodificada	84,3%	15,7%	100,0%
		% dentro de Mitrofanoff	100,0%	100,0%	100,0%
		% del total	84,3%	15,7%	100,0%

Pruebas de chi-cuadrado

	Valor	df	Significación asintótica (bilateral)	Significación exacta (bilateral)	Significación exacta (unilateral)
Chi-cuadrado de Pearson	0,089[a]	1	0,766		
Corrección de continuidad	0,000	1	1,000		
Razón de verosimilitud	0,091	1	0,763		
Prueba exacta de Fisher				1,000	0,565
Asociación lineal por lineal	0,087	1	0,768		
N de casos válidos	51				

a. 1 casillas (25,0%) han esperado un recuento menor que 5. El recuento mínimo esperado es 2,35.

Nefrectomía * **Mitrofanoff** – La prueba de chi-cuadrado NO ES VÁLIDA

Tabla cruzada

			Mitrofanoff		
			No	Sí	Total
Nefrectomía	No	Recuento	10	1	11
		% dentro de Nefrectomía	90,9%	9,1%	100,0%
		% dentro de Mitrofanoff	23,8%	12,5%	22,0%
		% del total	20,0%	2,0%	22,0%
	Unilateral	Recuento	14	4	18
		% dentro de Nefrectomía	77,8%	22,2%	100,0%
		% dentro de Mitrofanoff	33,3%	50,0%	36,0%
		% del total	28,0%	8,0%	36,0%
	Bilateral	Recuento	18	3	21
		% dentro de Nefrectomía	85,7%	14,3%	100,0%
		% dentro de Mitrofanoff	42,9%	37,5%	42,0%
		% del total	36,0%	6,0%	42,0%
Total		Recuento	42	8	50

Pruebas de chi-cuadrado

	Valor	df	Significación asintótica (bilateral)
Chi-cuadrado de Pearson	0,955[a]	2	0,620
Razón de verosimilitud	0,971	2	0,615
Asociación lineal por lineal	0,039	1	0,844
N de casos válidos	50		

a. 3 casillas (50,0%) han esperado un recuento menor que 5. El recuento mínimo esperado es 1,76.

Nefrectomía dicotomizada * Mitrofanoff – No existen diferencias estadísticamente significativas entre los grupos

Tabla cruzada

			Mitrofanoff No	Mitrofanoff Sí	Total
Nefrectomía dicotomizada	No	Recuento	10	1	11
		% dentro de Nefrectomía dicotomizada	90,9%	9,1%	100,0%
		% dentro de Mitrofanoff	23,8%	12,5%	22,0%
		% del total	20,0%	2,0%	22,0%
	Sí	Recuento	32	7	39
		% dentro de Nefrectomía dicotomizada	82,1%	17,9%	100,0%
		% dentro de Mitrofanoff	76,2%	87,5%	78,0%
		% del total	64,0%	14,0%	78,0%
Total		Recuento	42	8	50

Pruebas de chi-cuadrado

	Valor	df	Significación asintótica (bilateral)	Significación exacta (bilateral)	Significación exacta (unilateral)
Chi-cuadrado de Pearson	0,501[a]	1	0,479		
Corrección de continuidad	0,059	1	0,809		
Razón de verosimilitud	0,557	1	0,455		
Prueba exacta de Fisher				0,666	0,430
Asociación lineal por lineal	0,491	1	0,484		
N de casos válidos	50				

a. 1 casillas (25,0%) han esperado un recuento menor que 5. El recuento mínimo esperado es 1,76.

Ampliación vesical * **Mitrofanoff** – Sí existen diferencias significativas entre los grupos. Podría actuar como factor de confusión

Tabla cruzada

			Mitrofanoff No	Mitrofanoff Sí	Total
Ampliación vesical	No	Recuento	38	2	40
		% dentro de Ampliación vesical	95,0%	5,0%	100,0%
		% dentro de Mitrofanoff	88,4%	25,0%	78,4%
		% del total	74,5%	3,9%	78,4%
	Sí	Recuento	5	6	11
		% dentro de Ampliación vesical	45,5%	54,5%	100,0%
		% dentro de Mitrofanoff	11,6%	75,0%	21,6%
		% del total	9,8%	11,8%	21,6%
Total		Recuento	43	8	51

Pruebas de chi-cuadrado

	Valor	df	Significación asintótica (bilateral)	Significación exacta (bilateral)	Significación exacta (unilateral)
Chi-cuadrado de Pearson	16,013[a]	1	0,000		
Corrección de continuidad[b]	12,486	1	0,000		
Razón de verosimilitud	13,273	1	0,000		
Prueba exacta de Fisher				0,001	0,001
Asociación lineal por lineal	15,699	1	0,000		
N de casos válidos	51				

a. 1 casillas (25,0%) han esperado un recuento menor que 5. El recuento mínimo esperado es 1,73.

***No hago la comparación con la variable de "tipo de ampliación" porque en este caso, aunque se agrupe, sigue sin ser posible obtener una prueba válida.*

Anticolinérgicos * Mitrofanoff – Sí existen diferencias estadísticamente significativas entre los grupos. Podría actuar como factor de confusión

Tabla cruzada

			Mitrofanoff		
			No	Sí	Total
Anticolinérgicos	No	Recuento	38	4	42
		% dentro de Anticolinérgicos	90,5%	9,5%	100,0%
		% dentro de Mitrofanoff	88,4%	50,0%	82,4%
		% del total	74,5%	7,8%	82,4%
	Sí	Recuento	5	4	9
		% dentro de Anticolinérgicos	55,6%	44,4%	100,0%
		% dentro de Mitrofanoff	11,6%	50,0%	17,6%
		% del total	9,8%	7,8%	17,6%
Total		Recuento	43	8	51

Pruebas de chi-cuadrado

	Valor	df	Significación asintótica (bilateral)	Significación exacta (bilateral)	Significación exacta (unilateral)
Chi-cuadrado de Pearson	6,834[a]	1	0,009		
Corrección de continuidad	4,449	1	0,035		
Razón de verosimilitud	5,529	1	0,019		
Prueba exacta de Fisher				0,025	0,025
Asociación lineal por lineal	6,700	1	0,010		
N de casos válidos	51				

a. 1 casillas (25,0%) han esperado un recuento menor que 5. El recuento mínimo esperado es 1,41.

CI * Mitrofanoff – Sí existen diferencias estadísticamente significativas entre los grupos. Podría actuar como factor de confusión

Tabla cruzada

			Mitrofanoff		
			No	Sí	Total
CI	No	Recuento	37	0	37
		% dentro de CI	100,0%	0,0%	100,0%
		% dentro de Mitrofanoff	86,0%	0,0%	72,5%
		% del total	72,5%	0,0%	72,5%
	Sí	Recuento	6	8	14
		% dentro de CI	42,9%	57,1%	100,0%
		% dentro de Mitrofanoff	14,0%	100,0%	27,5%
		% del total	11,8%	15,7%	27,5%
Total		Recuento	43	8	51

Pruebas de chi-cuadrado

	Valor	df	Significación asintótica (bilateral)	Significación exacta (bilateral)	Significación exacta (unilateral)
Chi-cuadrado de Pearson	25,076[a]	1	0,000		
Corrección de continuidad	20,942	1	0,000		
Razón de verosimilitud	25,191	1	0,000		
Prueba exacta de Fisher				0,000	0,000
Asociación lineal por lineal	24,585	1	0,000		
N de casos válidos	51				

a. 1 casillas (25,0%) han esperado un recuento menor que 5. El recuento mínimo esperado es 2,20.

<u>RVU evol</u> * Mitrofanoff – No existen diferencias estadísticamente significativas entre los grupos

Tabla cruzada

			Mitrofanoff No	Mitrofanoff Sí	Total
RVU evol	No	Recuento	29	3	32
		% dentro de RVU evol	90,6%	9,4%	100,0%
		% dentro de Mitrofanoff	72,5%	37,5%	66,7%
		% del total	60,4%	6,3%	66,7%
	Sí	Recuento	11	5	16
		% dentro de RVU evol	68,8%	31,3%	100,0%
		% dentro de Mitrofanoff	27,5%	62,5%	33,3%
		% del total	22,9%	10,4%	33,3%
Total		Recuento	40	8	48

Pruebas de chi-cuadrado

	Valor	df	Significación asintótica (bilateral)	Significación exacta (bilateral)	Significación exacta (unilateral)
Chi-cuadrado de Pearson	3,675[a]	1	0,055		
Corrección de continuidad	2,269	1	0,132		
Razón de verosimilitud	3,467	1	0,063		
Prueba exacta de Fisher				0,097	0,069
Asociación lineal por lineal	3,598	1	0,058		
N de casos válidos	48				

a. 1 casillas (25,0%) han esperado un recuento menor que 5. El recuento mínimo esperado es 2,67.

***No hago la comparación con la variable de "grado RVU" ni con "tratamiento RVU" porque la prueba de chi-cuadrado no es válida y no veo posibilidad de dicotomizarlas correctamente.*

<u>RVU al injerto</u> * Mitrofanoff – No existen diferencias estadísticamente significativas entre los grupos

Tabla cruzada

			Mitrofanoff No	Mitrofanoff Sí	Total
RVU al injerto	No	Recuento	18	3	21
		% dentro de RVU al injerto	85,7%	14,3%	100,0%
		% dentro de Mitrofanoff	48,6%	42,9%	47,7%
		% del total	40,9%	6,8%	47,7%
	Sí	Recuento	19	4	23
		% dentro de RVU al injerto	82,6%	17,4%	100,0%
		% dentro de Mitrofanoff	51,4%	57,1%	52,3%
		% del total	43,2%	9,1%	52,3%
Total		Recuento	37	7	44

Pruebas de chi-cuadrado

	Valor	df	Significación asintótica (bilateral)	Significación exacta (bilateral)	Significación exacta (unilateral)
Chi-cuadrado de Pearson	0,079[a]	1	0,778		
Corrección de continuidad	0,000	1	1,000		
Razón de verosimilitud	0,079	1	0,778		
Prueba exacta de Fisher				1,000	0,553
Asociación lineal por lineal	0,077	1	0,781		
N de casos válidos	44				

a. 2 casillas (50,0%) han esperado un recuento menor que 5. El recuento mínimo esperado es 3,34.

***No hago la comparación con la variable de "grado RVU injerto" porque la prueba de chi-cuadrado no es válida y no veo posibilidad de dicotomizarla correctamente.*

Diálisis previa * **Mitrofanoff** – No existen diferencias estadísticamente significativas entre los grupos

Tabla cruzada

			Mitrofanoff No	Mitrofanoff Sí	Total
Diálisis previa	No	Recuento	13	4	17
		% dentro de Diálisis previa	76,5%	23,5%	100,0%
		% dentro de Mitrofanoff	30,2%	50,0%	33,3%
		% del total	25,5%	7,8%	33,3%
	Sí	Recuento	30	4	34
		% dentro de Diálisis previa	88,2%	11,8%	100,0%
		% dentro de Mitrofanoff	69,8%	50,0%	66,7%
		% del total	58,8%	7,8%	66,7%
Total		Recuento	43	8	51

Pruebas de chi-cuadrado

	Valor	df	Significación asintótica (bilateral)	Significación exacta (bilateral)	Significación exacta (unilateral)
Chi-cuadrado de Pearson	1,186[a]	1	0,276		
Corrección de continuidad	0,463	1	0,496		
Razón de verosimilitud	1,131	1	0,287		
Prueba exacta de Fisher				0,416	0,243
Asociación lineal por lineal	1,163	1	0,281		
N de casos válidos	51				

a. 1 casillas (25,0%) han esperado un recuento menor que 5. El recuento mínimo esperado es 2,67.

***No hago la comparación con la variable de "tipo diálisis" porque la prueba de chi-cuadrado no es válida y no veo posibilidad de dicotomizarla correctamente.*

Tipo trasplante * **Mitrofanoff** – No existen diferencias estadísticamente significativas entre los grupos

Tabla cruzada

			Mitrofanoff No	Mitrofanoff Sí	Total
Tipo trasplante	Cadáver	Recuento	28	4	32
		% dentro de Tipo trasplante	87,5%	12,5%	100,0%
		% dentro de Mitrofanoff	65,1%	50,0%	62,7%
		% del total	54,9%	7,8%	62,7%
	Vivo	Recuento	15	4	19
		% dentro de Tipo trasplante	78,9%	21,1%	100,0%
		% dentro de Mitrofanoff	34,9%	50,0%	37,3%
		% del total	29,4%	7,8%	37,3%
Total		Recuento	43	8	51

Pruebas de chi-cuadrado

	Valor	df	Significación asintótica (bilateral)	Significación exacta (bilateral)	Significación exacta (unilateral)
Chi-cuadrado de Pearson	0,659[a]	1	0,417		
Corrección de continuidad	0,171	1	0,679		
Razón de verosimilitud	0,642	1	0,423		
Prueba exacta de Fisher				0,450	0,333
Asociación lineal por lineal	0,646	1	0,421		
N de casos válidos	51				

a. 1 casillas (25,0%) han esperado un recuento menor que 5. El recuento mínimo esperado es 2,98.

<u>Número de rechazos agudos</u> * Mitrofanoff – La prueba de chi-cuadrado NO ES VÁLIDA.

Tabla cruzada

			Mitrofanoff No	Mitrofanoff Sí	Total
Número rechazos agudos	0	Recuento	33	6	39
		% dentro de Número rechazos agudos	84,6%	15,4%	100,0%
		% dentro de Mitrofanoff	76,7%	75,0%	76,5%
		% del total	64,7%	11,8%	76,5%
	1	Recuento	8	2	10
		% dentro de Número rechazos agudos	80,0%	20,0%	100,0%
		% dentro de Mitrofanoff	18,6%	25,0%	19,6%
		% del total	15,7%	3,9%	19,6%
	3	Recuento	1	0	1
		% dentro de Número rechazos agudos	100,0%	0,0%	100,0%
		% dentro de Mitrofanoff	2,3%	0,0%	2,0%
		% del total	2,0%	0,0%	2,0%
	4	Recuento	1	0	1
		% dentro de Número rechazos agudos	100,0%	0,0%	100,0%
		% dentro de Mitrofanoff	2,3%	0,0%	2,0%
		% del total	2,0%	0,0%	2,0%
Total		Recuento	43	8	51

Pruebas de chi-cuadrado

	Valor	df	Significación asintótica (bilateral)
Chi-cuadrado de Pearson	0,515[a]	3	0,915
Razón de verosimilitud	0,817	3	0,845
Asociación lineal por lineal	0,112	1	0,738
N de casos válidos	51		

a. 5 casillas (62,5%) han esperado un recuento menor que 5. El recuento mínimo esperado es 0,16.

***Aquí se podría dicotomizar entre rechazos agudos sí/no, pero no sé si es relevante para vosotros. Avísame si quieres que lo haga.*

Complicaciones urológicas * Mitrofanoff – No existen diferencias estadísticamente significativas entre los grupos

Tabla cruzada

			Mitrofanoff		
			No	Sí	Total
Complicaciones urológicas	No	Recuento	42	8	50
		% dentro de Complicaciones urológicas	84,0%	16,0%	100,0%
		% dentro de Mitrofanoff	97,7%	100,0%	98,0%
		% del total	82,4%	15,7%	98,0%
	Sí	Recuento	1	0	1
		% dentro de Complicaciones urológicas	100,0%	0,0%	100,0%
		% dentro de Mitrofanoff	2,3%	0,0%	2,0%
		% del total	2,0%	0,0%	2,0%
Total		Recuento	43	8	51

Pruebas de chi-cuadrado

	Valor	df	Significación asintótica (bilateral)	Significación exacta (bilateral)	Significación exacta (unilateral)
Chi-cuadrado de Pearson	0,190[a]	1	0,663		
Corrección de continuidad	0,000	1	1,000		
Razón de verosimilitud	0,345	1	0,557		
Prueba exacta de Fisher				1,000	0,843
Asociación lineal por lineal	0,186	1	0,666		
N de casos válidos	51				

a. 2 casillas (50,0%) han esperado un recuento menor que 5. El recuento mínimo esperado es 0,16.

Complicaciones vasculares * **Mitrofanoff** – No existen diferencias estadísticamente significativas entre los grupos

Tabla cruzada

			Mitrofanoff No	Mitrofanoff Sí	Total
Complicaciones vasculares	No	Recuento	41	8	49
		% dentro de Complicaciones vasculares	83,7%	16,3%	100,0%
		% dentro de Mitrofanoff	95,3%	100,0%	96,1%
		% del total	80,4%	15,7%	96,1%
	Sí	Recuento	2	0	2
		% dentro de Complicaciones vasculares	100,0%	0,0%	100,0%
		% dentro de Mitrofanoff	4,7%	0,0%	3,9%
		% del total	3,9%	0,0%	3,9%
Total		Recuento	43	8	51

Pruebas de chi-cuadrado

	Valor	df	Significación asintótica (bilateral)	Significación exacta (bilateral)	Significación exacta (unilateral)
Chi-cuadrado de Pearson	0,387[a]	1	0,534		
Corrección de continuidad	0,000	1	1,000		
Razón de verosimilitud	0,698	1	0,404		
Prueba exacta de Fisher				1,000	0,708
Asociación lineal por lineal	0,380	1	0,538		
N de casos válidos	51				

a. 2 casillas (50,0%) han esperado un recuento menor que 5. El recuento mínimo esperado es 0,31.

<u>Tipo inmunosupresión</u> * Mitrofanoff – No existen diferencias estadísticamente significativas entre los grupos

Tabla cruzada

			Mitrofanoff		
			No	Sí	Total
Tipo inmunosupresión	Cyc	Recuento	22	4	26
		% dentro de Tipo inmunosupresión	84,6%	15,4%	100,0%
		% dentro de Mitrofanoff	51,2%	50,0%	51,0%
		% del total	43,1%	7,8%	51,0%
	Azt	Recuento	21	4	25
		% dentro de Tipo inmunosupresión	84,0%	16,0%	100,0%
		% dentro de Mitrofanoff	48,8%	50,0%	49,0%
		% del total	41,2%	7,8%	49,0%
Total		Recuento	43	8	51

Pruebas de chi-cuadrado

	Valor	df	Significación asintótica (bilateral)	Significación exacta (bilateral)	Significación exacta (unilateral)
Chi-cuadrado de Pearson	0,004[a]	1	0,952		
Corrección de continuidad	0,000	1	1,000		
Razón de verosimilitud	0,004	1	0,952		
Prueba exacta de Fisher				1,000	0,626
Asociación lineal por lineal	0,004	1	0,952		
N de casos válidos	51				

a. 2 casillas (50,0%) han esperado un recuento menor que 5. El recuento mínimo esperado es 3,92.

ITUs de repetición * **Mitrofanoff** – Sí existen diferencias estadísticamente significativas entre los grupos. Podría actuar como factor de confusión

Tabla cruzada

			Mitrofanoff No	Mitrofanoff Sí	Total
ITUs de repetición	No	Recuento	34	1	35
		% dentro de ITUs de repetición	97,1%	2,9%	100,0%
		% dentro de Mitrofanoff	79,1%	12,5%	68,6%
		% del total	66,7%	2,0%	68,6%
	Sí	Recuento	9	7	16
		% dentro de ITUs de repetición	56,3%	43,8%	100,0%
		% dentro de Mitrofanoff	20,9%	87,5%	31,4%
		% del total	17,6%	13,7%	31,4%
Total		Recuento	43	8	51

Pruebas de chi-cuadrado

	Valor	df	Significación asintótica (bilateral)	Significación exacta (bilateral)	Significación exacta (unilateral)
Chi-cuadrado de Pearson	13,883[a]	1	0,000		
Corrección de continuidad	10,964	1	0,001		
Razón de verosimilitud	13,300	1	0,000		
Prueba exacta de Fisher				0,001	0,001
Asociación lineal por lineal	13,611	1	0,000		
N de casos válidos	51				

a. 1 casillas (25,0%) han esperado un recuento menor que 5. El recuento mínimo esperado es 2,51.

<u>Edad al trasplante (en años)</u> * Mitrofanoff - No existen diferencias entre los grupos. Prueba de la t de Student con un nivel de significación de 0,942.

	Mitrofanoff	N	Media	Desv. estándar
Edad al trasplante (años)	No	43	7,0271	4,99820
	Sí	8	6,9063	4,09012
